# Intracellular Bacillary Burden Reflects a Burst Size for *Mycobacterium tuberculosis* In Vivo

**DOI:** 10.1371/journal.ppat.1003190

**Published:** 2013-02-21

**Authors:** Teresa Repasy, Jinhee Lee, Simeone Marino, Nuria Martinez, Denise E. Kirschner, Gregory Hendricks, Stephen Baker, Andrew A. Wilson, Darrell N. Kotton, Hardy Kornfeld

**Affiliations:** 1 Department of Medicine, University of Massachusetts Medical School, Worcester, Massachusetts, United States of America; 2 Department of Microbiology and Immunology, University of Michigan Medical School, Ann Arbor, Michigan, United States of America; 3 Department of Cell Biology, University of Massachusetts Medical School, Worcester, Massachusetts, United States of America; 4 Department of Quantitative Health Science, University of Massachusetts Medical School, Worcester, Massachusetts, United States of America; 5 Pulmonary Center, Boston University School of Medicine, Boston, Massachusetts, United States of America; McGill University, Canada

## Abstract

We previously reported that *Mycobacterium tuberculosis* triggers macrophage necrosis in vitro at a threshold intracellular load of ∼25 bacilli. This suggests a model for tuberculosis where bacilli invading lung macrophages at low multiplicity of infection proliferate to burst size and spread to naïve phagocytes for repeated cycles of replication and cytolysis. The current study evaluated that model in vivo, an environment significantly more complex than in vitro culture. In the lungs of mice infected with *M. tuberculosis* by aerosol we observed three distinct mononuclear leukocyte populations (CD11b^−^ CD11c^+/hi^, CD11b^+/lo^ CD11c^lo/−^, CD11b^+/hi^ CD11c^+/hi^) and neutrophils hosting bacilli. Four weeks after aerosol challenge, CD11b^+/hi^ CD11c^+/hi^ mononuclear cells and neutrophils were the predominant hosts for *M. tuberculosis* while CD11b^+/lo^ CD11c^lo/−^ cells assumed that role by ten weeks. Alveolar macrophages (CD11b^−^ CD11c^+/hi^) were a minority infected cell type at both time points. The burst size model predicts that individual lung phagocytes would harbor a range of bacillary loads with most containing few bacilli, a smaller proportion containing many bacilli, and few or none exceeding a burst size load. Bacterial load per cell was enumerated in lung monocytic cells and neutrophils at time points after aerosol challenge of wild type and interferon-γ null mice. The resulting data fulfilled those predictions, suggesting a median in vivo burst size in the range of 20 to 40 bacilli for monocytic cells. Most heavily burdened monocytic cells were nonviable, with morphological features similar to those observed after high multiplicity challenge in vitro: nuclear condensation without fragmentation and disintegration of cell membranes without apoptotic vesicle formation. Neutrophils had a narrow range and lower peak bacillary burden than monocytic cells and some exhibited cell death with release of extracellular neutrophil traps. Our studies suggest that burst size cytolysis is a major cause of infection-induced mononuclear cell death in tuberculosis.

## Author Summary

Macrophages patrol the lung to ingest and destroy inhaled microbes. *Mycobacterium tuberculosis*, the bacteria causing tuberculosis, can survive within macrophages and use them as a protected environment for growth. Macrophages by themselves are poorly equipped to kill *M. tuberculosis* but may undergo programmed cell death (apoptosis) to limit bacterial replication. Virulent *M. tuberculosis* has evolved the capacity to inhibit macrophage apoptosis, thereby protecting the replication niche. In previous studies we showed that upon reaching a threshold intracellular number (burst size), virulent *M. tuberculosis* kills macrophages by necrosis and escapes for spreading infection. The present study was designed to test whether this mechanism seen in vitro operates during pulmonary tuberculosis in vivo. The distribution of *M. tuberculosis* numbers inside lung phagocytes of mice with tuberculosis conformed to predictions based on the burst size hypothesis, as did the appearance of dying cells. We identified four different types of phagocytes hosting intracellular *M. tuberculosis*. The distribution of *M. tuberculosis* load within individual phagocytes and between different types of phagocyte changed over the course of tuberculosis disease. These studies reveal the complexity of host defense in tuberculosis that must be considered as new therapies are sought.

## Introduction

Natural infection with *Mycobacterium tuberculosis* (Mtb) occurs by inhalation, followed by invasion of resident alveolar macrophages that provide the major initial replication niche for the pathogen. Macrophages infected with Mtb in vitro may die with primarily apoptotic or necrotic features [1]; the cell death mode most relevant to TB disease in vivo remains undefined. A widely held paradigm is that macrophage apoptosis promotes host defense in TB while necrosis favors spreading infection. We previously reported that the cytolytic activity of Mtb correlates with intracellular bacillary burden in macrophages, increasing dramatically at a threshold load of ∼25 bacilli per macrophage [2]. At high intracellular burden, Mtb triggers a primarily necrotic death dependent on bacterial genes regulated by the PhoPR 2-component system [3]. Our in vitro studies and data from other groups suggest that virulent Mtb strains suppress apoptosis of host macrophages [4]–[8] and grow to a threshold burden [2], [9] whereupon necrosis is triggered as an exit mechanism analogous to the burst size of lytic viruses.

In the present study, we investigated whether the necrotic death described for Mtb-infected macrophages in vitro is relevant to the fate of monocytic cells in the lung that become infected during the course of TB disease in vivo. Inhalation of Mtb is followed by the invasion of a small number of resident alveolar macrophages. We posit that within each infected macrophage, bacterial replication expands an initial low multiplicity of infection (MOI) to a burst size value. Once this threshold is exceeded, the liberated bacilli spread to naïve phagocytes. Successive rounds of invasion, replication and escape will result in a distribution of bacillary loads across the population of infected phagocytes. This model predicts that at any given time point after low dose aerosol challenge, phagocytes harboring 1–10 bacilli will outnumber those with higher bacillary loads, and that host cells containing ≥25 bacilli will be a distinct minority of infected cells. The model also predicts that with the induction of adaptive immunity (∼3 weeks after aerosol challenge), inhibition of Mtb replication will rescue many infected cells with a low bacillary burden from progressing to burst size. This will increase the proportion of cells containing 1–10 bacilli while heavily infected cells will die and be replaced at a low rate thereby reducing the proportion of cells containing ≥25 bacilli.

To test those predictions we enumerated acid fast bacilli (AFB) per cell in whole lung leukocytes and bronchoalveolar lavage (BAL) cells harvested from mice after low dose aerosol infection with Mtb Edrman. The distribution of AFB burden in monocytic cells harvested from wild type (WT) C57BL/6 mice followed the predicted pattern. Analysis of interferon-γ knockout (GKO) mice with TB also conformed to the predicted effects of unrestricted Mtb replication on the distribution of bacillary loads per cell. The morphology of heavily infected monocytic cells isolated from the lungs of mice with TB exhibited features similar to those seen after high MOI challenge in vitro: nuclear condensation without fragmentation and loss of cell membrane integrity without cell swelling or apoptotic vesicle formation. Taken together, these results support the burst size hypothesis for TB in vivo.

During the course of these experiments it became apparent that the diversity of cell types hosting Mtb in vivo adds an additional layer of complexity to TB pathogenesis. Neutrophils were major Mtb host cells 2–3 weeks post infection (p.i.) but rarely contained >10 AFB per cell. We also observed differences in the distribution of Mtb between three subpopulations of mononuclear leukocytes classified by CD11b and CD11c expression. The proportion of infected host cells differed between these subpopulations, and the proportions changed dynamically between 4 and 10 weeks p.i. The relative permissiveness or restriction of intracellular Mtb replication as well as the regulation of host cell fate may differ for each of these phagocytic cell types with implications for host defense, immune pathology and latent TB infection.

## Results

### Dynamic shifts of monocytic cells hosting Mtb in evolving pulmonary TB

Alveolar macrophages are the overwhelming majority of leukocytes in the normal alveolar space and they are considered the primary leukocyte type initially infected by inhaled Mtb in vivo. Any investigation of the burst size hypothesis for TB must consider the complexity of the in vivo environment and the potential for Mtb to invade a diverse spectrum of phagocytic cells in the lung [10], [11]. To study the distribution of Mtb within subpopulations of monocytic cells, we harvested lungs of WT mice 4 and 10 weeks after aerosol challenge with Mtb Erdman. Whole lung CD45^+^ leukocytes were sorted on the basis of CD11b and CD11c expression. Previous reports have extensively characterized subpopulations of leukocytes in lung tissues of mice with TB [11]–[18]. In accordance with those studies, we classified alveolar macrophages (AM) as CD11b^−^ CD11c^+/hi^, recruited monocyte/macrophages (RM) as CD11b^+/lo^ CD11c^lo/−^ and myeloid dendritic cells (mDC) as CD11b^+/hi^ CD11c^+/hi^ (Fig. S1A). For clarity we refer to AM, RM and mDC populations in this report based on CD11b and CD11c expression, recognizing that this might not invariably correspond to functional identity. We evaluated additional cell surface markers including Ly-6G (Fig. S1B), CD115, F4/80 and MHC class II (not shown) but these provided no additional useful discriminatory information. Uninfected mice were sampled for comparison to the TB group at both time points (Table S1).

**Figure 1:**
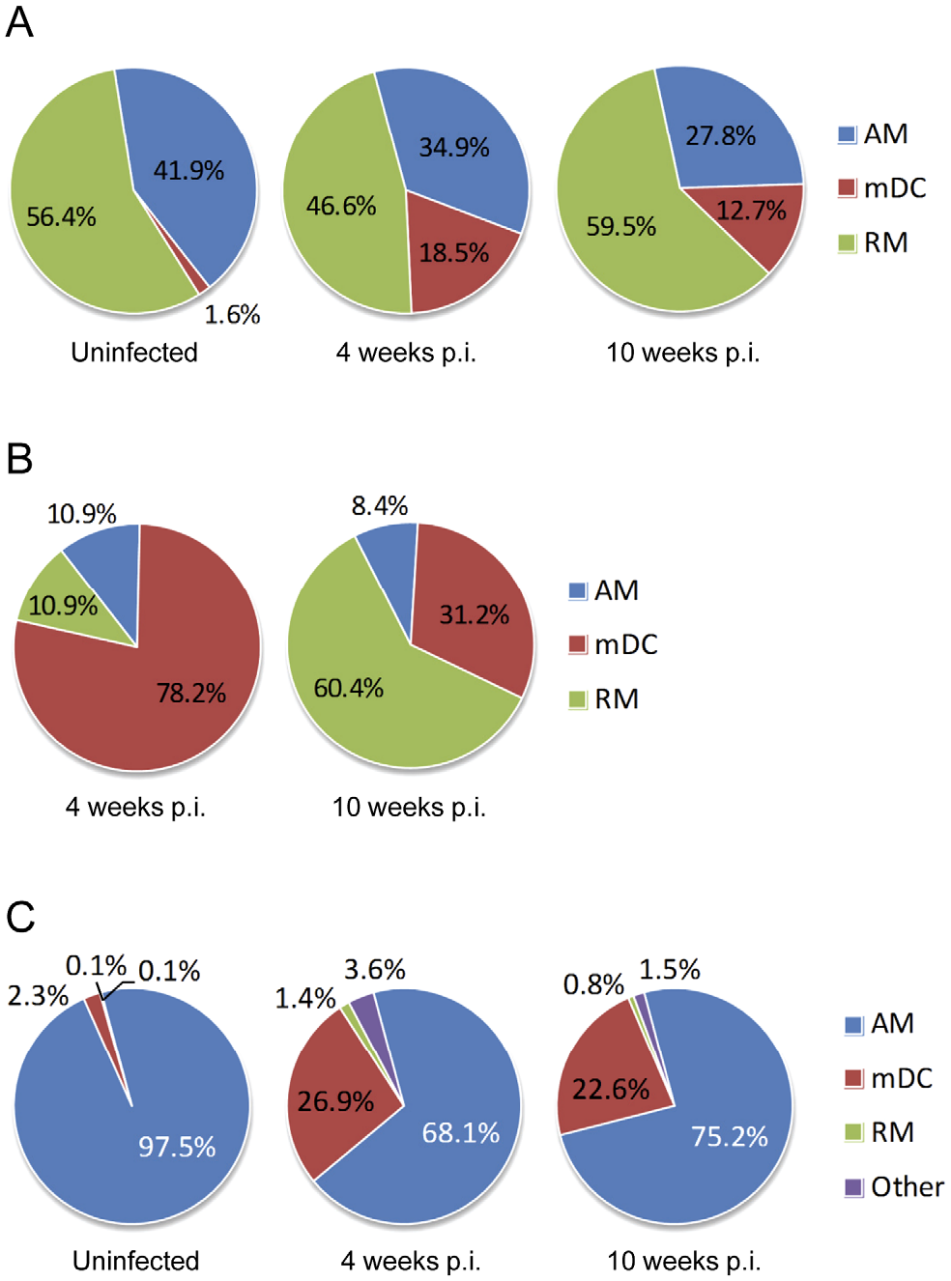
Distribution of Mtb infection within monocytic cell populations in the lung. Whole lung leukocytes were harvested from groups of mice with TB (4 and 10 weeks p.i.) and compared to uninfected controls. Monocytic cells were sorted into AM, RM and mDC as described in *Materials and Methods*. Ziehl-Neelsen staining was performed on cytospin preparations of sorted populations. (A) The proportion of AM, RM and mDC within the total lung monocytic cell population of uninfected mice and mice with pulmonary TB. (B) The proportion of AM, RM and mDC containing any AFB in mice with pulmonary TB. (C) The proportion of GFP-labeled lung leukocytes, GFP^+^ AM, RM and mDC, in uninfected mice and mice with TB. Lung leukocytes within the airspace were transduced by tracheal instillation of WT mice with CMV-GFP-W. After 8 weeks, one group of GFP-transduced mice was challenged by aerosol with 300 CFU of Mtb Erdman delivered to the lung. The category *Other* comprised cells that could not be categorized as AM, RM or mDC based on light scatter characteristics and CD11b/CD11c staining. By light microscopy, cells in the *Other* category included a small number of neutrophils that may have acquired GFP by efferocytosis, as well of cells with monocytic appearance that had very high intracellular Mtb burden and features of cell death. Monocytic cell subsets were classified by surface immunostaining as alveolar macrophages (AM; CD11b^−^ CD11c^+/hi^), recruited monocyte/macrophages (RM; CD11b^+/lo^ CD11c^lo/−^) and myeloid dendritic cells (mDC; CD11b^+/hi^ CD11c^+/hi^).

Mononuclear cells from uninfected lungs comprised a roughly equal proportion of AM and RM, with mDC a distinct minority at 1.6% (Fig. 1A). By 4 weeks p.i., the total number of mDC increased >40-fold and they expanded proportionately from 1.6% to 18.5% of monocytic cells. The distribution of monocytic cells changed slightly at 10 weeks p.i., with a moderate expansion of RM and modest contraction of the AM and mDC populations. Mtb-infected monocytic cells were enumerated by microscopy on cytospin slides with Ziehl-Neelsen staining (Table S2). After 4 weeks of TB disease, mDC were the predominant Mtb-infected cell type, representing 78.2% of total AFB^+^ monocytic cells (Fig. 1B). By 10 weeks p.i., the distribution of infected cells shifted, with RM becoming the primary Mtb host cells (60.4%), followed by mDC (31.2%). At both time points, AM represented approximately 10% of AFB^+^ monocytic cells. The relative propensity for different mononuclear leukocyte cell types to harbor Mtb was estimated based their representation in the total and AFB^+^ cells within the total population. On that basis, mDC were 13 and 18 times more likely to be infected with Mtb compared to AM or RM, respectively, at 4 weeks p.i.

### Resident alveolar macrophages are a minor niche for Mtb replication

Resident AM, by far the predominant leukocytes in the airspace of normal lungs, are the first phagocytes to become infected by Mtb following inhalation of droplet nuclei. In the absence of infection or inflammation they are extremely long-lived, non-replicating cells with negligible replacement by bone marrow-derived leukocytes [19]. To investigate the impact of pulmonary TB on resident AM, we labeled these cells by tracheal instillation of a replication incompetent, VSVG-pseudotyped, GFP-expressing lentivirus (CMV-GFP-W) as previously described [20]. Labeling efficiency is typically 30–40% of BAL cells, with no transduction of epithelial cells or parenchymal leukocytes. After an early loss of transduced cells in the first several weeks, the number of GFP^+^ cells stabilizes at 20–30% of BAL cells for up to 2 years. To track the fate of resident AM in TB, mice were transduced with intratracheal CMV-GFP-W and rested for 8 weeks to allow the GFP^+^ cell population to stabilize. One group of transduced mice was then infected with Mtb Erdman by aerosol while control transduced mice remained uninfected. Sets of Mtb-infected and control mice were sacrificed 4 weeks and 10 weeks after infection of the TB group and whole lung leukocytes were isolated for analysis by flow cytometry (Fig. 1C).

In uninfected mice, GFP^+^ leukocytes comprised 97% AM, 2.8% mDC and <0.3% RM or other cells (Table S3) reflecting the typical composition of leukocytes in airspace under basal conditions. After 4 weeks of TB disease, the total number of GFP^+^ cells was little changed from baseline but the proportion AM among all GFP^+^ cells fell to 68% while mDC increased to 27% of GFP^+^ cells. Thus, ∼22% of AM shifted their surface phenotype to one resembling mDC. Whether this represents induction of CD11b surface expression on cells that retain AM properties, or if these cells convert into functional mDC remains to be determined. The phenotypic shift of resident AM contributed only ∼3% of the total increase in lung mDC at 4 weeks p.i., indicating increased mDC population mostly resulted from differentiation of monocytic cells newly recruited to the lung. Comparison of total GFP^+^ cells counts between groups was limited by variation in labeling efficiency but the data suggest a trend after 10 weeks of TB for a modest loss of AM that were resident in the lung prior to infection. We found no GFP^+^ leukocytes containing AFB at 4 or 10 weeks p.i., suggesting little or no horizontal spread of infection through the resident AM population present in the lung prior to infection. Most if not all of the AM accounting for ∼10% of Mtb-infected monocytic cells at 4 and 10 weeks p.i. (Fig. 1B) were recruited to the lung after infection. We conclude that resident AM may be critical host cells for intracellular infection by inhaled Mtb, but only for the first round of bacillary replication to burst size. Subsequently, the rapidly expanding number of bacilli shifts to phagocytes newly recruited to the lung, most of which do not differentiate into AM.

### Enumeration of intracellular bacilli in pulmonary TB

**Figure 2:**
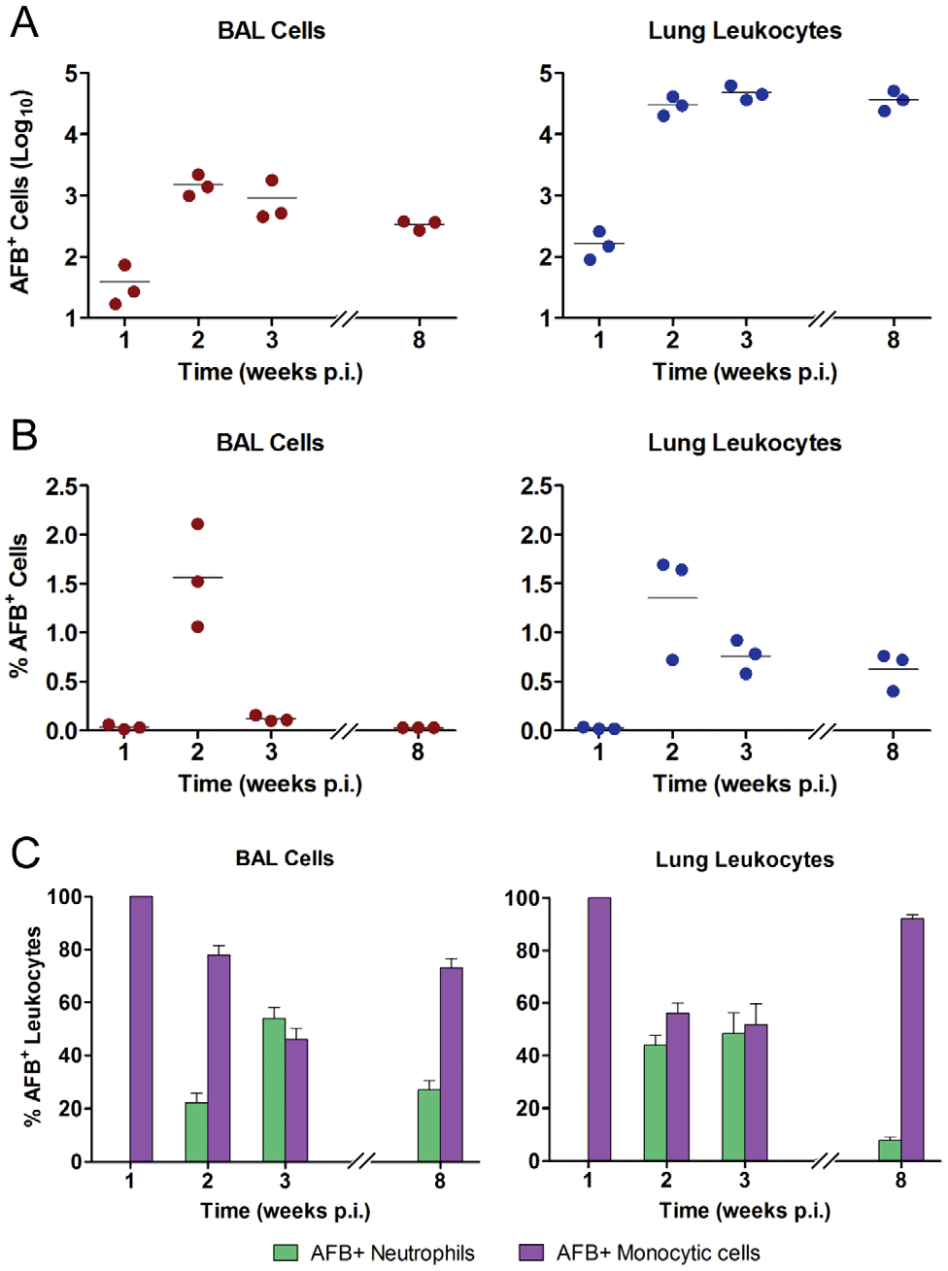
Kinetics of intracellular Mtb growth in vivo. BAL cells and whole lung leukocytes were harvested at 1, 2, 3 and 8 weeks after aerosol infection of mice with 300 CFU Mtb Erdman delivered to the lung. Cells from both sources were counted and cytospin preparations were made for Ziehl-Neelsen staining. (A) The total number of Mtb-infected (AFB^+^) cells was derived by multiplying the % AFB^+^ cells by the total number of cells in each sample. Results for individual mice are presented as log_10_ total AFB^+^ cells, with the line representing the mean. (B) The proportion of Mtb-infected phagocytes within the total sample was counted at each time point and expressed as % AFB^+^ cells. (C) Neutrophils infected with Mtb were identified by their typical nuclear morphology on Ziehl-Neelsen stained cytospin slides of BAL cells and whole lung leukocytes. The relative proportion of AFB in neutrophils versus monocytic phagocytes (AM, RM and mDC) is expressed as mean % AFB^+^ leukocytes ± SD from one representative experiment. All in vivo experiments were repeated twice.

To investigate the in vivo relevance of a burst size model for macrophage cell death in TB, we challenged C57BL/6 mice with virulent Mtb Erdman by aerosol set to deliver ∼300 CFU to the lung. Lung cells were subsequently harvested in different sets of infected mice exclusively by BAL or by enzymatic digestion to isolate whole lung leukocytes. Cells were immobilized onto slides by cytocentrifugation and intracellular bacilli were visualized with Ziehl-Neelsen staining. The percent and total number of AFB^+^ cells was enumerated at time points between 1 and 8 weeks p.i. In parallel with the characteristic kinetics of lung bacillary load in the aerosol TB model, the total number of AFB^+^ cells increased logarithmically until reaching a plateau value between 2 and 3 weeks p.i. (Fig. 2A). When compared to whole lung leukocytes, the total number of AFB^+^ BAL cells declined by 8 weeks p.i., likely reflecting a loss of airspace available to lavage. In contrast, the total number of AFB^+^ lung leukocytes remained stable between 2 and 8 weeks p.i., reflecting the major site of TB disease more accurately than BAL at later time points. The proportion of BAL cells and lung leukocytes infected with Mtb peaked around 1.5% at 2 weeks p.i. and then declined, owing to the recruitment of naïve leukocytes to the infected lung (Fig. 2B). Light microscopy allowed reliable identification of neutrophils that were therefore counted separately from monocytic cells. Mononuclear leukocyte subsets, comprising AM, RM and mDC, are not visually distinguishable. AFB^+^ neutrophils were not seen in BAL or lung leukocytes at 1 week p.i. but equaled monocytic cells as hosts for Mtb at weeks 2 and 3, the period of greatest bacillary expansion (Fig. 2C).

**Figure 3:**
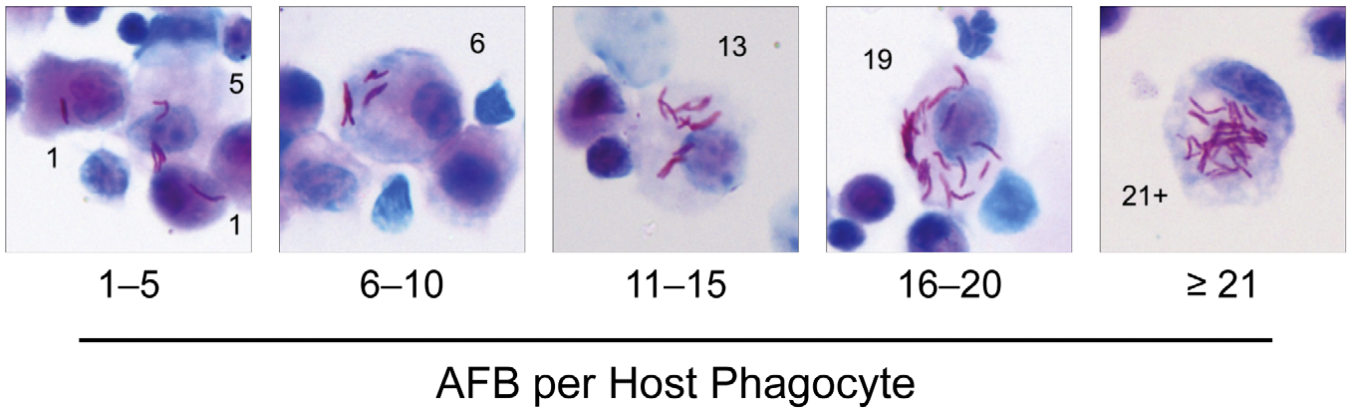
Enumeration of intracellular Mtb in lung phagocytes. BAL cells and whole lung leukocytes were isolated from C57BL/6 mice 2 weeks after aerosol challenge with Mtb Erdman. Ziehl-Neelsen stained cytospin preparations were used to visualize and count intracellular AFB by light microscopy. Representative photomicrographs show examples of infected cells along with AFB counts as indicated (magnification, 400×).

**Figure 4:**
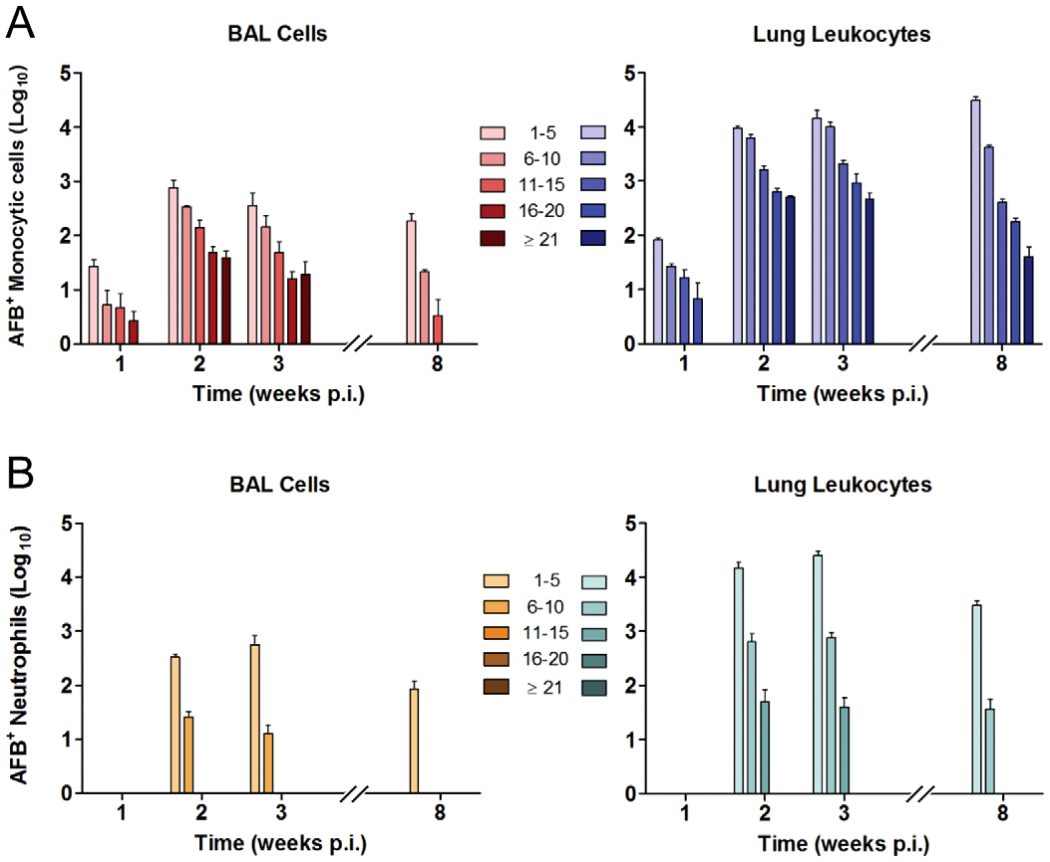
Distribution of intracellular bacillary load in lung phagocytes changes over time after aerogenic Mtb infection. The number of AFB per cell was counted in BAL cells and lung leukocytes harvested at the indicated times after aerosol challenge with Mtb Erdman. Across all time points, Mtb burden per cell was interrogated in a total 5.7×10^6^ individual phagocytes, with counts grouped into bins of 1–5, 6–10, 11–15, 16–20, or ≥21 AFB. Results are expressed as mean log_10_ AFB^+^ monocytic cells (A) or neutrophils (B) ± SD within each bin, counted in BAL cells or in whole lung leukocytes as indicated. All bins were compared at each time point and between time points as described in *Materials and Methods*. Statistically significant differences (p<0.05) are not indicated on the figure for the sake of clarity. Among statistically significant differences, monocytic cells with 1–10 AFB outnumbered cells with >10 AFB at all time points p.i., the proportion of monocytic cells containing >15 AFB was lower at week 8 than earlier time points, and the distribution of AFB loads was significantly different in neutrophils compared to monocytic cells.

At each time point after aerosol challenge, AFB per neutrophil or monocytic cell was counted in individual cells. AFB counts were grouped into five bins (1–5, 6–10, 11–15, 16–20 and ≥21) with the top bin reflecting the highest burden that we could reliably count to ±2 AFB (Fig. 3). Consistent with the distribution of intracellular bacillary loads predicted for a burst size model, the number of AFB^+^ monocytic cells containing 1–5 AFB was greater than cells with higher bacillary loads at all time points (Fig. 4A). Also consistent with the burst size hypothesis, the proportion of AFB^+^ monocytic cells harboring ≥21 bacilli peaked during the period of logarithmic Mtb replication at weeks 1 to 3 p.i. and then declined in parallel with induction of adaptive immunity. We previously reported an in vitro burst size of ∼25 bacilli for Mtb-infected bone marrow derived macrophages (BMDM) [2]. In the present study, the bin of whole lung monocytic cells containing ≥21 AFB peaked at 2.8% of all AFB^+^ monocytic cells by 2 weeks p.i. and then fell to 0.1% by 8 weeks p.i. (Fig. S2). We posit that cells with high burden reflect Mtb replication towards burst size, which dropped by a factor of 10 following the induction of adaptive immunity. The absolute number of heavily infected cells declined between 3 and 8 weeks p.i., indicating that cells dying after reaching burst size were being replaced at a reduced rate as host immunity limited Mtb replication. Monocytic cells estimated to contain up to 50 or more AFB were very rarely seen, representing 0.017% of AFB^+^ cells at 3 weeks p.i. Most such cells appeared nonviable, with faintly stained cytoplasm and bacilli breaching the plasma membrane (Fig. S3A).

We counted AFB per cell in cytospin preparations of flow-sorted monocytic cell types but found few cells containing >10 AFB. This was inconsistent with results obtained with BAL cells or lung leukocytes that were cytocentrifuged directly onto the slides without further processing. Speculating that heavily burdened phagocytes progressing to necrosis were fragile and unable to withstand the stress of flow sorting, we examined the sorted population of non-viable cells defined by forward-scatter and side-scatter characteristics. Ziehl-Neelsen stained cytospins of those samples revealed much higher numbers of AFB in these dead and dying cells than in the sorted populations of viable cells (Fig. S3B). Collectively, these results support the concept of a mononuclear leukocyte burst size for virulent Mtb in vivo with a median value in the range of 20–40 bacilli. Of interest, while lung leukocytes with ≥16 AFB declined by 84.5% between 3 and 8 weeks p.i., some high burden cells were still seen at the later time point, well after the induction of adaptive immunity when total lung bacillary load is held stable. Our findings in TB contrast with a report that viable macrophages isolated from the footpads of *M. leprae*-infected athymic *nu*/*nu* mice contained an average of 120 AFB per cell [21]. Unlike Mtb, *M. leprae* is not cytolytic for macrophages in vitro even at MOI 100 [22].

In contrast to the distribution of Mtb in monocytic cells, neutrophils containing >15 AFB were not seen at any time point, and neutrophils with 11–15 AFB were identified only at 2 and 3 weeks p.i. (Fig. 4B). This corresponds to the period of logarithmic Mtb expansion in the lung and the peak number of the most heavily infected monocytic cells. These results suggest that neutrophils are recruited to the vicinity of necrotic monocytic cells and acquire bacilli at low to moderate MOI. The data also imply that neutrophils may be poor hosts for Mtb replication, presumably owing to their limited lifespan. Alternatively, neutrophils could be subject to burst size cytolysis with a lower threshold value or some other cell death mode in the context of TB.

### Mtb replication dynamics in IFN-γ-deficient mice

**Figure 5:**
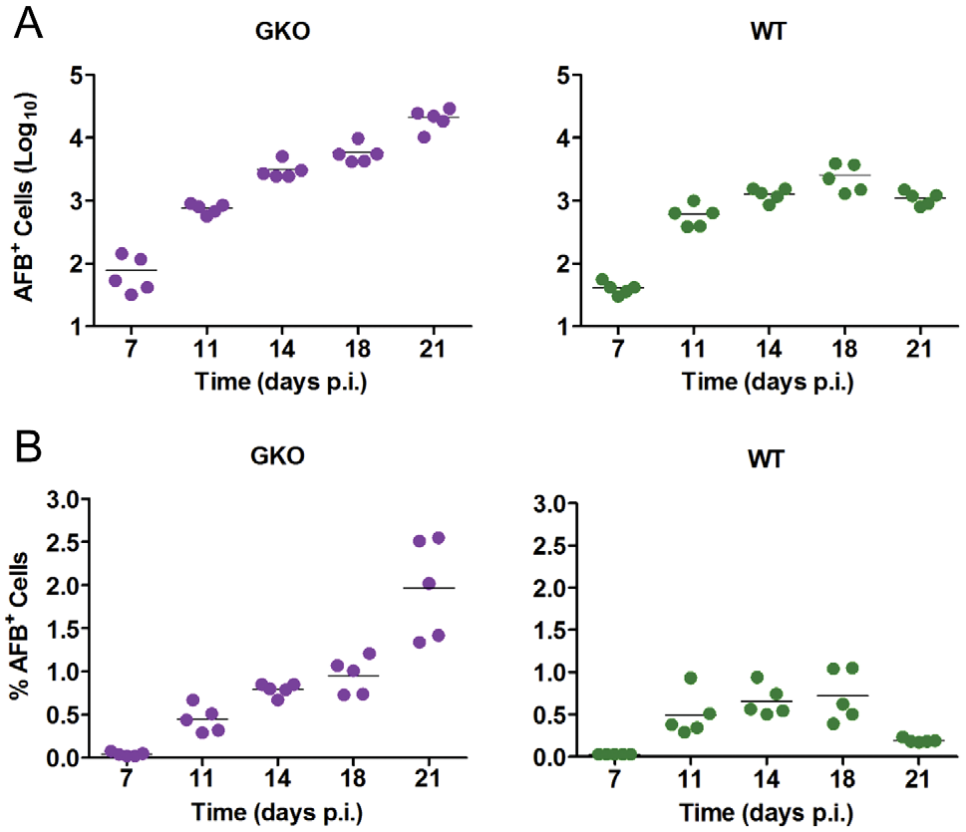
Kinetics of Mtb growth in IFN-γ-deficient mice. BAL cells were harvested from WT and GKO mice 7, 11, 14, 18 and 21 days after aerosol challenge with 100 CFU Mtb Erdman delivered to the lung. Total cells from both sources were counted and cytospin preparations were made for Ziehl-Neelsen staining. (A) The total number of Mtb-infected (AFB^+^) cells was derived by multiplying the % AFB^+^ cells by the total number of cells in each sample. Results for individual mice are presented as log_10_ total AFB^+^ cells, with the line representing the mean. (B) The proportion of Mtb-infected phagocytes within the total sample was counted at each time point and expressed as % AFB^+^ cells with the bar indicating the mean. Data were analyzed as detailed in *Materials and Methods*. The number of AFB^+^ BAL cells from GKO mice was significantly different (p<0.05) from WT on days 14, 18 and 21 p.i. The % AFB^+^ BAL cells from GKO mice were significantly different from WT on day 21 p.i. Data are representative from one experiment with five mice per group. In vivo experiments were repeated twice.

**Figure 6:**
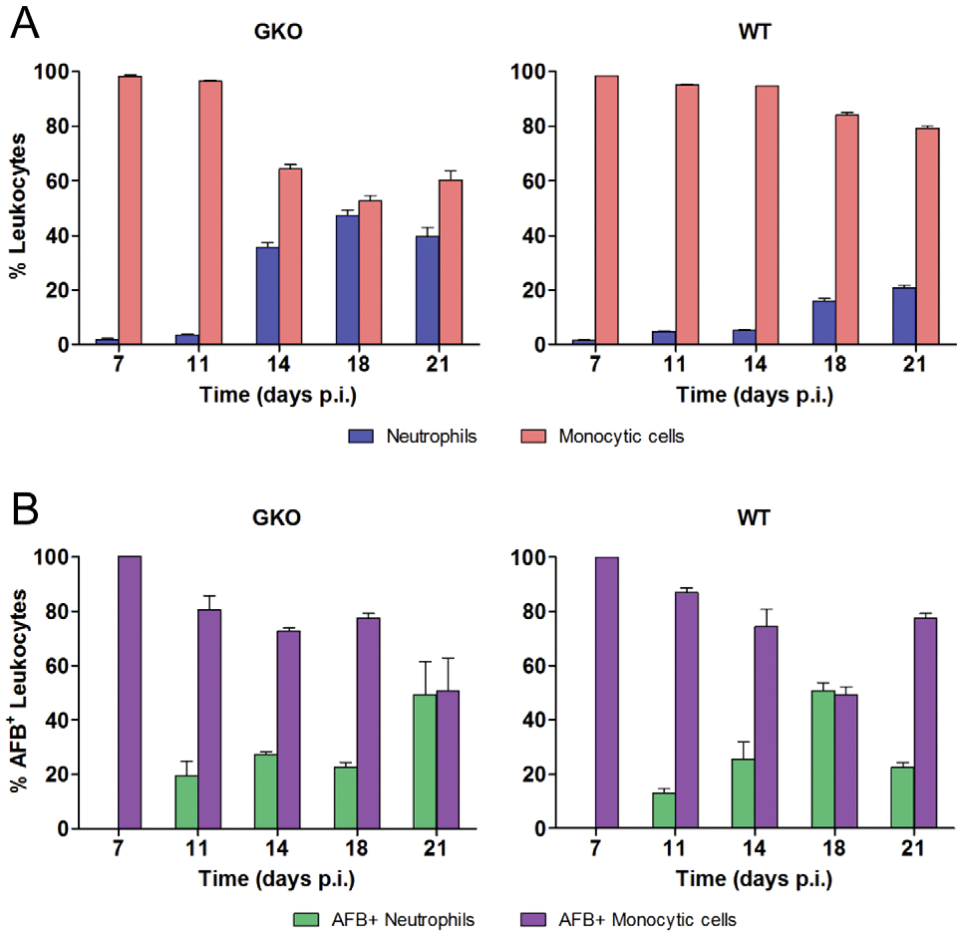
Lung leukocyte populations and distribution of intracellular Mtb in WT and GKO mice. (A) The relative proportion of neutrophils and monocytic cells in BAL from WT and GKO mice following aerogenic infection with Mtb Erdman was determined by light microscopy. Results are expressed as mean % cells of either type ± SD at the indicated time points. All groups were compared within and between all time points. GKO mice had a significantly higher proportion of neutrophils to monocytic cells compared to WT at 14, 18 and 21 days p.i. (B) The distribution of Mtb infection between neutrophils and monocytic cells was determined by counting AFB^+^ cells of both types on Ziehl-Neelsen stained cytospins. Results are expressed as mean % AFB^+^ ± SD. There was a statistically significant difference in the number of AFB^+^ neutrophils between WT and GKO mice on day 18 p.i. There was also a significant increase in the number of AFB^+^ neutrophils at day 21 p.i. for GKO mice compared to earlier time points.

Interferon (IFN)- γ plays a critical role in protective immunity against Mtb by activating macrophages to limit bacterial replication [23]. After aerosol challenge of GKO mice, lung Mtb burden increases logarithmically until death by 4–6 weeks p.i. We delivered 100 CFU of Mtb Erdman to WT and GKO mice and then harvested BAL cells for cytospin and Ziehl-Neelsen staining at five time points from 7 to 21 days. As expected, the total number of AFB^+^ BAL cells increased progressively in GKO mice while in WT mice it was held to a plateau value after day 18 p.i. (Fig. 5). It was recently proposed that IFN-γ limits neutrophil recruitment to the lung in the transition from innate to adaptive immunity in TB [24]. Consistent with that report, neutrophils represented a higher proportion of BAL cells in GKO compared to WT mice with TB (Fig. 6A). While neutrophils represented only 16% of BAL leukocytes in WT mice at 18 days p.i., they accounted for ∼50% of AFB^+^ cells at that time point (Fig. 6B). This suggests that despite the influence of IFN-γ on neutrophil trafficking, these cells are recruited to the immediate vicinity of Mtb infection where they may exert comparatively high phagocytic activity.

**Figure 7:**
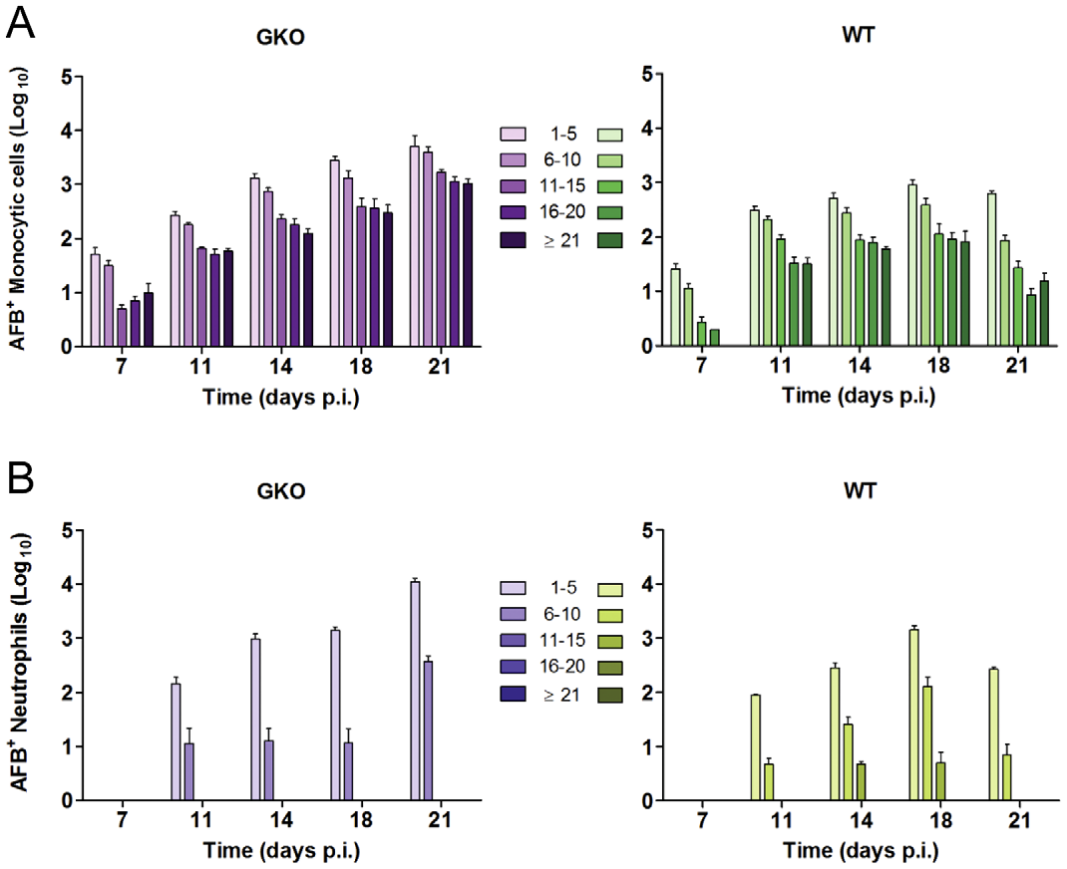
Distribution of AFB loads in lung monocytic cells and neutrophils from WT and GKO mice with TB. GKO and WT mice were challenged by aerosol with 100 CFU of Mtb Erdman delivered to the lung. BAL was performed 7, 11, 14, 18 and 21 days p.i. A total of 4.8×10^6^ Ziehl-Neelsen stained cells were counted. Numbers of AFB per cell were grouped into 5 bins as indicated and counted separately in monocytic cells (A) and neutrophils (B) from GKO and WT mice. Results are presented as mean log_10_ AFB^+^ cells in each bin ± SD. GKO mice had a greater number of AFB^+^ monocytic cells in all bins at 7 days p.i. and a significantly higher proportion of cells with ≥11 AFB compared to WT mice at that time point. The proportion of cells within each bin was similar between WT and GKO mice on days 11–18 p.i. but by day 21 p.i. the proportion of cells with ≥11 AFB fell significantly in WT compared to GKO mice. On day 21 p.i. the number of AFB^+^ neutrophils from GKO mice was significantly higher than earlier time points. In contrast, the number of AFB^+^ neutrophils from WT mice was significantly lower on day 21 than day 18 p.i.

Based on the burst size hypothesis, unrestricted intracellular Mtb replication in GKO mice is predicted to result in a persistently high proportion of heavily infected monocytic cells past the time point when WT mice start to restrict Mtb replication. Bacterial load per cell in BAL monocytic cells and neutrophils from GKO and WT mice was counted at each time point and tabulated in bins (Fig. 7). GKO mice had a higher proportion and total number of heavily infected cells on day 7 p.i. suggesting an innate IFN-γ response [25], [26] that limits Mtb replication before adaptive immunity is expressed. Prior to the full induction of adaptive immunity, Mtb replication in WT mice also proceeded at a rapid rate such that on day 14 p.i. the distribution of AFB loads was similar to that of GKO mice. As an effective IFN-γ-dominated adaptive immune response was expressed in WT mice, the proportion of heavily infected BAL cells declined. At day 21 p.i., the proportion AFB^+^ cells in the top three bins were significantly higher in GKO compared to WT mice. AFB^+^ cells containing 1–5 bacilli remained the most abundantly populated bin in GKO mice at all time points and cells containing >50 AFB were very rarely seen, as was the case in WT mice. We interpret the distribution of AFB loads in GKO compared to WT mice as supporting the burst size hypothesis and also indicating that IFN-γ has little if any direct influence on the burst size value.

### Cell death in vivo resembles high MOI necrosis in vitro

**Figure 8:**
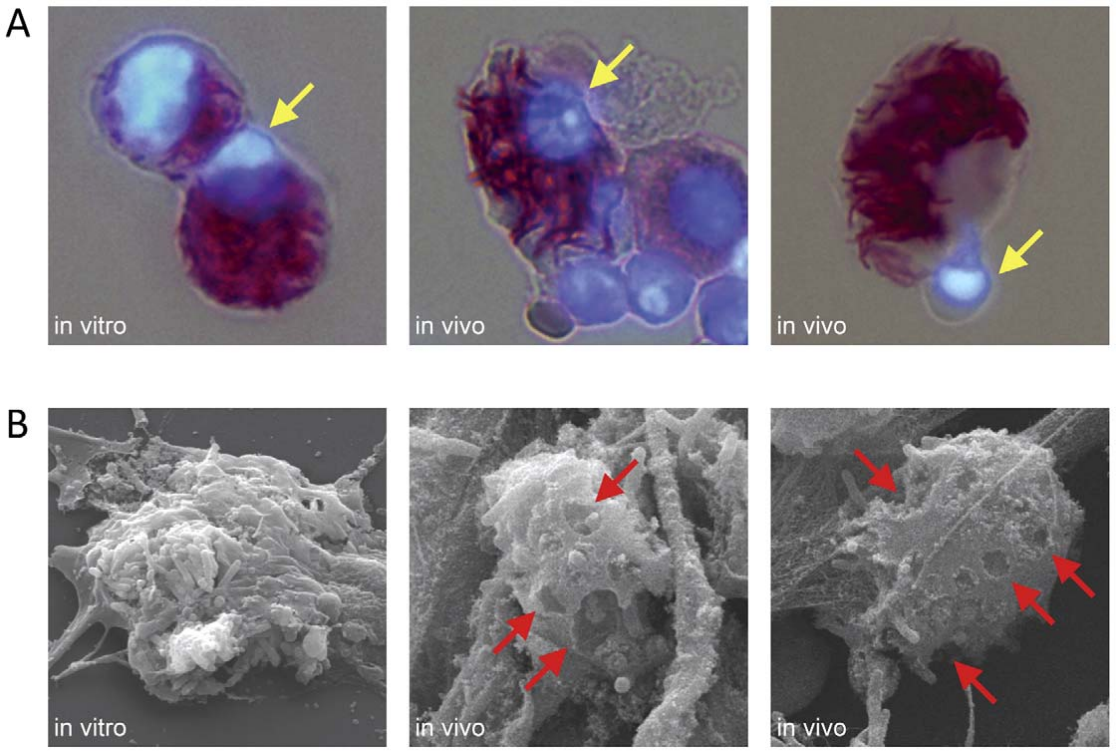
Morphology of macrophage cell death in pulmonary TB. (A) BMDM challenged with Mtb Erdman in vitro (MOI 25, 3 h) and BAL cells from WT mice challenged 3 weeks previously with a low aerosol dose of Mtb Erdman were stained with carbolfuchsin to visualize intracellular bacilli and stained with DAPI to visualize nuclear morphology (magnification, ×400). Heavily infected cells in both cases exhibited nuclear condensation without fragmentation (*yellow arrows*). (B) Representative scanning electron micrographs of BMDM infected with Mtb in vitro and BAL macrophages isolated from mice after 4 weeks of TB disease (magnification, ×5,000). Damaged cells in both cases exhibit disintegration of outer cell membranes (*red arrows*) with escape of intracellular bacilli. BAL cells were isolated from GKO mice 4 weeks after aerosol Mtb infection. Cytospin preparations were processed for Ziehl-Neelsen staining, immunostaining or scanning EM.

Macrophages challenged in vitro with Mtb at MOI ≥25 rapidly undergo an atypical, caspase-independent cell death dominated by lipolytic attack on lipid membranes [3]. This cell death mode has unique morphological features including nuclear condensation without fragmentation, and disintegration of lipid bilayers throughout the cell. In the present study, we compared the morphology of BAL cells harvested 3–4 weeks after low dose aerosol Mtb Erdman challenge to that of BMDM infected for 3 h in vitro with Mtb at MOI 25. Similar to the characteristic changes seen in vitro, monocytic cells from the lungs of mice with TB exhibited nuclear condensation and this was restricted to those cells with a high AFB burden (Fig. 8A). Nuclear fragmentation, a characteristic of caspase-mediated apoptosis, was not seen in >5×10^5^ DAPI-stained lung cells from mice with TB. The morphology of lung leukocytes having low numbers of intracellular bacilli was uniformly similar to the normal appearance of uninfected cells (Fig. S4).

Mtb-induced macrophage cytolysis in vitro is characterized by disintegration of mitochondrial, nuclear and plasma membranes without cell swelling or formation of apoptotic vesicles. To examine the ultrastructural features of infected lung leukocytes in the context of pulmonary TB, BAL cell cytospin preparations were visualized by scanning electron microscopy (EM) and compared to BMDM challenged with Mtb Erdman in vitro at MOI 25 (Fig. 8B). BAL cells isolated from mice with TB showed a similar pattern of injury to BMDM infected in vitro: plasma membrane damage and no evidence of budding vesicles or osmotic lysis. We also observed extrusion of chromatin through damaged nuclear membranes in dying BAL cells (Fig. S5), akin to results we previously reported with in vitro Mtb infection [3]. Taken together, these observations demonstrate consistent similarities between Mtb-induced cytolysis in vitro and in vivo.

### Mtb infection is associated with release of neutrophil extracellular traps in vivo

A high proportion of lung neutrophils were infected with Mtb after aerosol challenge and they were a significant host cell compartment for bacilli even after 8 weeks of TB disease. Compared to monocytic cells, neutrophils have a short lifespan in the lung in the absence of inflammation, which would seem to make them unproductive hosts for Mtb replication. Examining cytospin preparations of BAL cells from GKO mice, we observed large masses of amorphous extracellular material with numerous associated AFB (Fig. S6A). A combination of carbolfuchsin and DAPI stains indicated that these structures had a high content of extracellular DNA. Scanning EM revealed a network of extracellular fibrillar structures with adherent bacilli similar to the morphological features first described by Brinkmann and Zychlinsky [27]. Furthermore, the thread-like structures were abundantly studded with globular domains. The composition of NETs includes characteristic components including myeloperoxidase (MPO), neutrophil elastase and cleaved histones [28], [29]. Confocal scanning laser microscopy and immunostaining with antibodies against neutrophil elastase, MPO and histones showed co-localization of these molecules with extracellular DNA (Fig. S6B). Together, these data indicate the presence of neutrophil extracellular traps (NETs). We did not see NETs in cytospins from WT mice with TB. Release of NETs could be limited to conditions of uncontrolled Mtb replication in GKO mice but more likely occurs at a low frequency in WT mice that is undetectable by the methods we used. Furthermore, we observed Mtb-induced NETs in BAL from hypercholesterolemic ApoE null mice with TB (Fig. S6C). At the time of sampling p.i., these mice express comparable levels of IFN-γ with WT, but are unable to control bacterial replication and they develop severe neutrophilic lung inflammation [30]. Ramos-Kichik et al. [31] reported that Mtb induces NET release in vitro and that NETs trap the bacilli but are unable to kill them, in contrast to microbicidal activity of NETs against *Listeria monocytogenes*. We believe that our data from GKO and ApoE null mice are the first evidence for NET release in the context of TB disease in vivo and indicate that an extracellular population of bacilli may be adherent to NETs in the lung.

## Discussion

We examined leukocytes from the lungs of mice infected with Mtb by aerosol to test a model of burst size cytolysis suggested by prior in vitro studies. The distribution of Mtb load in monocytic cells was skewed such that most AFB^+^ cells contained few bacilli while a minority had a high bacillary load. The morphology of heavily infected cells mirrored that seen with Mtb-induced necrosis in vitro; they appeared nonviable, with condensed nuclei and disrupted plasma and nuclear membranes. We interpret these findings as consistent with burst size cytolysis at median threshold in the range of 20–40 AFB. That value is close to the burst size reported for in vitro infection of BMDM [2]. The comparison of WT and GKO mice demonstrated that by limiting Mtb replication to burst size, IFN-γ promotes the survival of monocytic host cells with a sublethal bacillary burden. A similar phenomenon was reported for BMDM in vitro, where virulent Mtb strains introduced at low MOI grew rapidly and caused necrosis but cytolysis was prevented when Mtb replication was inhibited by exogenous IFN-γ [9]. Despite an effective immune response in WT mice, some monocytic cells with >15 AFB were present at 8 weeks p.i., accounting for 0.6% of all AFB^+^ cells at that time. This implies ongoing Mtb replication in a limited population of monocytic cells balanced by microbicidal activity in as yet unknown compartments during the period of “stationary persistence” that is not reflected by an increase in total lung CFU. A similar conclusion was reached by Gill et al. [32] in a study that employed in silico modeling and in vivo experiments using Mtb transformed with an unstable plasmid replication clock.

Virulent Mtb strains inhibit host-protective apoptotic death of infected macrophages [4]–[8], permitting optimal replication before spreading to other cells. Transit between replication niches requires time and incurs risk for bacilli that may be trapped in the extracellular environment, subjected to antimicrobial activities, or taken up by phagocytes that do not support replication. Delaying host cell death for 3–5 Mtb doublings should accelerate the increase in total lung bacillary load in the critical period prior to the induction of adaptive immunity. Lacking any means to manipulate burst size in biological experiments, we took advantage of an existing computational model to test the effects of different burst size values on Mtb accumulation in the lung. This in silico agent-based model replicates the interplay between host and pathogen, taking multiple variables into account over three biological scales: molecular, cellular and tissue in a 2 mm×2 mm section of lung. The model captures burst size by setting a value to the maximum carrying capacity of Mtb per macrophage, above which the macrophage bursts, releasing viable bacilli to infect naïve cells. We varied burst size values in the computational model to 10, 20, 30, 40, and 50, keeping all other parameter values fixed in order to analyze the effects of this isolated change. To account for stochastic variability, each experiment was run 20 times (equivalent to using 20 mice for each time point).

Figure S7 shows five time courses of total bacterial counts for each burst size assumption. By day 20, a burst size >20 resulted in a significantly greater increase in total bacteria as compared to smaller burst sizes. For burst sizes 10 and 20, a peak is reached at day 20 and a lower steady state is achieved as adaptive immunity is expressed in the lung. For burst sizes >20, the Mtb count either stabilizes at the peak (burst size 30) or trends up at a slower rate (burst sizes 40 and 50). Overall, increasing the burst size resulted in higher bacterial loads, consistent with advantage for the pathogen with burst size >20. While providing independent support for the burst size hypothesis, the model has several limitations. In its current iteration, only macrophages are considered as hosts for Mtb and it assumes that every bacillus liberated from a dying macrophage invades a different new host cell at MOI 1. Our animal data show that AM, RM, mDC and neutrophils all harbor Mtb and these cell types likely differ in their capacity to support or inhibit bacillary replication. The biological data also suggest that the efficiency of Mtb escape and reinfection of new host cells is lower than the model assumes since we saw neutrophils (cells unlikely to support multiple rounds of Mtb replication) harboring up to 15 AFB. We also frequently observed Mtb in clumps that would deliver multiple bacilli if ingested by a single phagocyte (Fig. S3C). Insights from the in vivo TB study presented here will be applied to future refinements of the agent-based model.

We identified three discrete monocytic cell populations hosting Mtb, in general agreement with prior reports [10]–[12]. We recognize that monocytic cells hosting Mtb in the lung may be even further subclassified [14], and that functional heterogeneity between individual cells of the same surface phenotype is likely. Cells classified as mDC were increased in number and proportion by 4 weeks p.i. and were favored hosts for Mtb at that time. This increase was due mostly to recruitment, with a minor contribution from phenotypic shift of AM resident in the lung prior to aerosol Mtb challenge as demonstrated by lentiviral GFP labeling. By 10 weeks p.i., the predominant AFB^+^ monocytic cells were RM. The basis for that switch is presently unknown but might relate to the reported increase of GM-CSF and decrease of M-CSF in the lung over time after aerosol Mtb infection, which correlates with reduced DC-like cell surface markers and increased foamy macrophages [13]. The extent to which mDC and RM defined by surface phenotype differ at a functional level is unknown. They might differentially restrict or permit Mtb replication, differ in their response to IFN-γ, or differ in susceptibility to cytolysis. Ryan et al. [33] reported that Mtb induces non-apoptotic death of human peripheral blood-derived DC, with some features similar to those of murine BMDM challenged at high MOI in vitro. Our in vivo data imply that tissue mDC are subject to burst size cytolysis, but this has not been directly tested in vitro. IFN-γ-activated BMDM restrict Mtb replication in vitro more effectively than activated bone marrow-derived DC [34]. If that difference holds in vivo, it would favor RM survival with a sub-lethal Mtb load and the preferential accumulation of these cells in the lung over time p.i. Loss of heavily infected monocytic cells during the process of flow sorting prevented us from comparing the distribution of AFB loads within purified populations of mDC, RM and AM. We are exploring alternative approaches to generate such data.

We confirmed that neutrophils are also major Mtb host cells in vivo, albeit with a narrower range of AFB load than monocytic cells. As a proportion of all AFB^+^ cells, neutrophils were major Mtb hosts in the period of logarithmic increase of total lung bacillary load. The proportion of neutrophils with relatively high burden (6–15 AFB) also peaked at 2–3 weeks p.i. in WT mice. In GKO mice, total lung Mtb burden and infected neutrophils increased logarithmically until death. Cytokines play a major role in the positive and negative regulation of neutrophil trafficking to the lung in TB. Nandi and Behar reported that coincident with the induction of adaptive immunity, IFN-γ inhibits neutrophil accumulation in part by reducing Th17 differentiation [24]. We found that neutrophils accounted for half of all AFB^+^ leukocytes on day 18 p.i. in WT mice, despite being a distinct minority of total lung leukocytes at that time. This indicates that neutrophils are recruited to the proximity of necrotic cells at foci of Mtb infection in the lung. We propose that death-associated molecular patterns (DAMPs) released from monocytic cells undergoing Mtb-induced necrosis contribute to neutrophil recruitment and activation in TB. In that regard, we previously described neutrophil-rich inflammation and a high frequency of cell death in pulmonary TB lesions of diabetic and hypercholesterolemic mice that express IFN-γ to an equal or greater extent than mice without metabolic disorders [30], [35], [36], and we showed that HMGB1 is released in the course of Mtb burst size cytolysis [37]. Despite their recruitment to TB lesions and phagocytosis of bacilli, neutrophils are unlikely to host multiple rounds of Mtb replication before dying by spontaneous apoptosis or by NETosis.

In summary, our data support a burst size model for Mtb cytolysis in vivo. The features of this atypical necrotic death that we characterized in vitro are favorable for an exit mechanism in vivo. Mtb-induced cell death occurs at a threshold intracellular burden, it liberates the bacilli free of apoptotic vesicles, and it has little impact on Mtb viability. We did not find any DAPI-stained cells with fragmented nuclei or signs of apoptotic vesicle formation by scanning EM of BAL or lung leukocytes in the present study. While classical apoptosis has clearly been demonstrated in TB, our data suggest that burst size necrosis is a common fate for Mtb-infected monocytic cells in vivo. The burst size model logically fits into the pathogenesis of TB but our results highlight complex host-pathogen interactions. Resident AM are a transient niche for Mtb immediately after inhalation, but bacilli rapidly move into cells with surface phenotypes of mDC or RM, preferentially infecting the former early in disease and then shifting to the latter during stationary persistence. Neutrophils avidly acquire bacilli in the 3 week interval of logarithmic increase in total lung bacterial load. Their trafficking may be regulated in part by DAMPs released through burst size cytolysis of Mtb-infected cells. Neutrophils may promote host defense in the transition from innate to adaptive immunity [38], [39], but play a detrimental role if they accumulate in excess, as occurs with poorly controlled TB in mice and in humans [36], [40], [41]. NETs lack antimicrobial activity against Mtb in vitro [31]. Their potential to reduce Mtb viability in vivo is presently unknown. In neutrophilic TB lesions, NETs might promote lung injury as they were shown to do in a mouse influenza model [42]. It is interesting to consider what role NETs could play in forming a milieu that supports extracellular persistence of Mtb in necrotic lung lesions. A refined understanding of host-pathogen interactions in TB will require analysis of unique Mtb interactions with each of these phagocyte types in vivo, using cells isolated from the tuberculous lung.

## Materials and Methods

### Ethics statement

Experiments with animals were conducted according to the National Institutes of Health guidelines for housing and care of laboratory animals and performed under protocols approved by the Institutional Animal Care and Use Committee and the Institutional Biosafety Committee at The University of Massachusetts Medical School (UMMS).

### Mice

C57BL/6 WT, IFN-γ^−/−^ (B6.129S7-Ifng^tm1Ts^/J) knockout mice (#2287), and ApoE^−/−^ were purchased from The Jackson Laboratory. Mice were housed in specific pathogen-free environment at Animal Medicine facility of UMMS.

### Bacterial strain and Mtb infection

Mtb Erdman was used for in vitro and aerosol infections. Bacterial stocks for experiments were prepared as described previously [3]. For in vitro infections, BMDM were generated as previously described [2] and plated in Lab-Tek tissue culture chamber slides (Nalge Nunc International) at a density of 2×10^5^ cells per well, or in 24-well cell culture plates at 5×10^5^ cells per well in complete DMEM. Cells were infected with Mtb Erdman (MOI 25, 3 h, 37°C), washed with PBS and then overlaid with fresh complete DMEM. For aerosol infections, mice were exposed to Mtb in a Glas-Col Inhalation Exposure System set to deliver ∼100 CFU or ∼300 CFU to the lung. For each experiment, 2 mice were sacrificed 24 hours p.i. to verify the delivered dose as described.

### Cell preparation

Lung leukocytes were isolated as previously described [35]. Briefly, mice were sacrificed and lungs were perfused through the heart with PBS. Excised lungs were minced and digested with 150 U/ml collagenase IV and 60 U/ml DNase (Sigma-Aldrich; 45 min, 37°C). Processed tissues were filtered using a 40 µm cell strainer and treated with Gey's Solution (Sigma-Aldrich). BAL cells were collected by flushing lungs three times with 0.75 ml PBS containing 0.2% BSA and 0.2 mM EGTA, and added to1.0 ml of 20% FBS in PBS and placed immediately on ice. BAL fluid was washed in PBS and treated with Gey's Solution. Whole lung leukocytes and BAL cells prepared in this manner were fixed in 1.5% paraformaldehyde for overnight at 4°C. Fixed cell suspensions were washed, re-suspended in PBS and stored in 4°C. Cell counts were determined using a hemocytometer.

### Intracellular bacterial enumeration

BAL cells and lung leukocytes were harvested from Mtb infected mice at predetermined time points. Slides were prepared using cytocentrifugation to immobilize 1×10^5^ cells per slide (Thermo Electron Corporation). Cytospin slides were heat-fixed for Ziehl-Neelsen staining kit (TB Stain Kit ZN, BD Diagnostic Systems) following manufacturer's protocol. Stained slides were visualized using a Nikon Eclipse E400 Microscope and photomicrographs were obtained with a Nikon DS-Ri1 camera using NIS-Elements Microscope Imaging Software. Individual cells were interrogated for intracellular bacteria by counting AFB encased or surrounded by cellular membrane. Accurately counting intracellular AFB was reliable at low bacillary burden but became progressive more difficult in high burden cells with clumped bacilli. AFB counts were grouped into five bins: 1–5, 6–10, 11–15, 16–20, and ≥21. Cells were identified as monocytic cells (comprising AM, RM, mDC) or neutrophils based on nuclear morphology. AFB counts were tallied separately for these two categories.

### Acid-fast, DAPI and immunostaining

To examine the nuclear morphology, cytospin slides were heat fixed and submerged in TB Carbolfuchsin ZN (BD Diagnostic Systems). Slides were heated in microwave oven for two consecutive intervals of 15 sec separated by 2 min at room temperature and then gently rinsed under running distilled water and decolorized with TB Decolorizer (BD Diagnostic Systems). Slides were rinsed again and then stained with 0.5 g/ml of 4′,6′-diamidino-2-phenylindole, dihydrochloride (DAPI) staining for 2 min. After a final rinse, slides were dried and cover slips mounted with ProLong Gold Antifade reagent (Invitrogen).

For immunostaining, BAL cells were affixed to Cell-Tak (BD Biosciences) treated cytospin slides and blocked with 3% BSA and 10% goat serum in PBS. Cells were stained with primary antibodies, 1∶50 myeloperoxidase (LS Bio) and 1∶50 histone H2B (Santa Cruz Biotechnology) or 1∶50 neutrophil elastase (Calbiochem). Fluorescent anti-rabbit antibodies conjugated to Alexa Fluor 488, 568, 594 or 647 (Invitrogen) were used as secondary antibodies. Cells were mounted and stained with DAPI with Prolong Gold Antifade Reagent with DAPI (Invitrogen). Analysis of immunostained cells were performed with confocal scanning laser microscopy (SP2 AOBS Leica) and images were captured using LCS software.

### Flow cytometry and analysis of lung cell population

BAL cells and lung leukocytes were washed and incubated with CD16/CD32 mAb (BD Biosciences) to block Fc binding. Cells were then stained with the following mAb purchased from eBioscience (San Diego, CA): eFluor450–anti-CD11b (M1/70); phycoerythrin–anti-CD11c (N418); allophycocyanin–anti-CD45 (30-F11); APC-eFluor780-anti-Ly-6G (RB6-8C5); and Live/Dead Fixable Dead Cell Stain Kit by Invitrogen. An LSRII flow cytometer (BD Biosciences) was used for acquisition and data were analyzed with FlowJo software (TreeStar). Unless otherwise stated, gating was set to exclude dead cells and lymphocyte populations in forward/side scatter graph and to include singlet cells in a dot plot of pulse area against pulse height. Gating on viable cells, we defined resident AM as CD11b^−^ CD11c^+/hi^ cells, RM as CD11b^+/lo^ CD11c^lo/−^ and mDC as CD11b^+/hi^ CD11c^+/hi^ (Fig. S1). Cells were sorted utilizing BD FACSAria Cell Sorter (BD Biosciences) with the same gating strategies used for flow cytometry. Subsets of sorted cell populations were collected and affixed onto cytospin slides for Ziehl-Neelsen staining and enumeration of intracellular AFB by light microscopy.

### GFP labeling of resident alveolar macrophages

A replication incompetent, VSVG-pseudotyped, lentivirus expressing GFP under the control of a CMV promoter (CMV-GFP-W) was used to transduce resident lung leukocytes. The vector was created using a 5-plasmid transfection method previously described [20], [43]. Briefly, 293T cells were transfected with the pHAGE backbone lentiviral vector together with 4 expression vectors encoding the packaging proteins Gag-Pol, Rev, Tat, and the G protein of the vesicular stomatitis virus (VSV-G). To transduce lung cells, the viral titer was adjusted to 5×10^9^/ml in DMEM with 10% FBS and mixed with lipofectamine 2000 (Invitrogen) at a ratio of 100∶5 (v∶v) on ice for 15–30 min. Mice were then infected by tracheal instillation of 5×10^7^ virions in a volume of 50 ul.

### Scanning electron microscopy

Samples of non-adherent cells infected with Mtb were processed by first preparing microscope slides with Cell-Tak (BD Biosciences). Cell suspensions were added to treated and dried slides by cytocentrifugation and allowed to bond to the Cell-Tak. The cells on the slides were fixed by immersion in 2% paraformaldehyde (v/v)/2.5% glutaraldehyde (v/v) in 0.1 M Na cacodylate-HCl buffer (pH 7.2) overnight at 4°C. The next day the fixed samples were washed three times in 0.5 M Na cacodylate-HCl buffer (pH 7.0) and then post-fixed for 1 hr in 1% osmium tetroxide (w/v) in the same buffer. Following post-fixation, samples were dehydrated through a graded series of ethanol to two changes of 100% ethanol and critical point dried in liquid CO_2_. The microscope slides were cut to remove the excess glass, mounted onto aluminum stubs with silver conductive paste and then coated with carbon (1 nm) and then sputter coated with gold/palladium (4 nm). Specimens were then examined using an FEI Quanta 200 FEG MK II scanning electron microscope.

### Computational model of TB infection in the lung

A 2-dimensional (2D) agent-based model (ABM) framework developed [44]–[46] for spatially characterizing the mechanisms of immunity in the lung during TB infection was used to test the burst size concept. The virtual environment reflects a 2 mm×2 mm section of lung parenchyma represented as a 100×100 2D grid with micro-compartments scaled to the approximate size of a macrophage (∼20 µm). A virtual low dose infection is triggered by one infected macrophage (M_I_), with one intracellular Mtb. The ABM describes interactions between intracellular and extracellular Mtb, various states of macrophages (resting, infected, chronically infected and activated), T cell populations including CD4^+^, CD8^+^ and regulatory T cells along with major cytokines tumor necrosis factor-α and IFN-γ and chemokine effector molecules (e.g., CCL2, CCL5, CXCL9/10/11). Each immune cell's behavior adapts based on its environment and its interactions with other immune cells and Mtb. As infection progresses, Mtb is tracked continuously. Extracellular Mtb proliferation follows a logistic growth function (48 h doubling time) within a single micro-compartment with a given carrying capacity while intracellular Mtb follows an exponential growth curve with a doubling time of 24 h. Intracellular Mtb doubling time is set to 72 h after adaptive immunity appears at the infection site (i.e., 20 days p.i.).With the current mechanisms already present in our model that can affect Mtb levels, we captured the process of burst size cytolysis by setting a maximum carrying capacity for a chronically infected macrophage. If the intracellular bacterial load exceeds this threshold, the macrophage bursts releasing viable bacteria into the extracellular space. The model allows a user defined parameter value for this threshold, labeled as *burst size*. For this study, we varied the burst sizes from 10 to 50 while maintaining fixed values for the remaining parameters to analyze affects of different burst sizes on the total lung bacterial burden.

Uncertainty and sensitivity analysis (U/SA) [47] has been used in this model to ensure that the selected parameter values influencing outcomes of infection (e.g. clearance, containment or dissemination) are in accordance with known dynamics. The results of U/SA analysis provide constructive evaluation of the critical processes and mechanisms suggesting strategies for model reduction, questions requiring additional in vivo experimentation, and to generate alternative hypotheses if burst size is not supported by model results [48].

### Statistical analysis

Unless otherwise stated, data from independent experiments are shown as mean ± SD or SEM. Comparisons between groups were evaluated with Student *t-*test using GraphPad Prism. Differences in the distribution of AFB load in frequency bins obtained from experiments with GKO and WT mice evaluated using analysis of variance for mixed model [49] with Restricted Maximum Likelihood (REML) algorithm [50] for fitting the model. Load data were transformed using natural logarithms to better approximate normally distributed errors, an assumption of the mixed model ANOVA. The distributional characteristics of the data were evaluated using the Kolmogorov-Smirnov goodness of fit test [51] upon model residuals. A p value<0.05 was regarded as statistically significant.

In the computational model, standard unidirectional *t*-test, with heteroscedasticity assumption (i.e., different variability between groups) was used to test statistically significant differences (p<0.05) between time course predictions with different burst sizes at different time points.

## Supporting Information

Figure S1 **Gating scheme for immunophenotyping lung leukocyte populations.** (A) Gating steps to analyze lung cell populations include gating on singlets as having a similar height and area (FSC-A/FSC-H), on live and myeloid cells (FSC-A/SSC-A), and on CD45^+^ hematopoietic cells (SSC-A/CD45). Each cell population is defined based on CD11b/CD11c staining profile: alveolar macrophages (AM, CD11b^−^ CD11c^+/hi^), myeloid DC (mDC, CD11b^+/hi^ CD11c^+/hi^), neutrophils (PMN, CD11b^+/hi^ CD11c^−^), and recruited monocyte-macrophages (RM, CD11b^+/lo^ CD11c^lo/−^). (B) Schematic illustration of the gating strategy of lung leukocytes. A stepwise gating strategy was applied to lung cell populations gating on myeloid phagocytes, singlets, live cells, and CD45^+^ cells. CD45^+^ cells are divided into four cell types based on CD11b/CD11c expression profiles. Each cell type is further analyzed by CD11b/Ly-6G staining profiles. Curved quadrants were set according to fluorescence-minus-one. This figure indicates that the cell population defined by CD11b/CD11c profile is identical to those by CD11b/Ly-6G. (C) Gating on GFP expressing lentivirus-transduced leukocytes. Cells were transduced in vivo by tracheal instillation of CMV-GFP-W lentivirus as described in Materials and Methods. This approach exclusively labels cells within the airspace of the lung. After an 8 week rest period to allow the GFP^+^ cell population to stabilize, BAL cells were recovered for CD11b/CD11c immunostaining and flow cytometry. Based on a CD45/GFP scatter graph, GFP is expressed exclusively by CD45^+^ cells. Most of GFP^+^ cells comprise AM (CD11b^−^ CD11c^+/hi^) consistent with the fact that the vast majority of leukocytes in the alveolar space under basal conditions are resident AM. A very small proportion of GFP^+^ cells fall in the mDC gate.
(PDF)

Figure S2 **Change in the proportion of intracellular bacillary load in monocytic cells.** AFB per cell was counted in cytospin samples of whole lung leukocytes harvested 1, 2, 3 and 8 weeks after aerosol challenge with Mtb Erdman. Mtb burden per monocytic cell (comprising AM, RM, mDC) was counted and stratified into the indicated bins of 1–5, 6–10, 11–15, 16–20, or ≥21. Results are expressed as mean % AFB^+^ monocytic cells within each bin ± SD at the indicated time points. Statistical analysis described in *Materials and Methods* confirmed a significantly different distribution of AFB load in high bins at 8 weeks p.i. as compared to earlier time points.
(PDF)

Figure S3 **Cells heavily burdened with Mtb appear nonviable.** (A) Lung leukocytes were isolated from WT mice 2 weeks after aerosol challenge with Mtb Erdman. Cytospin preparations were made and Ziehl-Neelsen stain was used to visualize and count intracellular AFB by light microscopy at 400× magnification. Photomicrographs show examples of heavily infected cells with ∼50 intracellular AFB. (B) Whole lung leukocytes harvested 4 weeks after aerosol Mtb challenge were prepared for cell sorting. Cytospin preparations were made from the sorted population of “dead” cells defined by lower forward-scatter and higher side-scatter characteristics. AFB where visualized with Ziehl-Neelsen staining (magnification, ×400). (C) Lung leukocytes from WT mice with 3 weeks of TB disease were processed by cytocentrifugation and Ziehl-Neelsen staining. The image shows clumps of AFB associated with dead cell remnants barely capable of retaining dye (magnification, ×400).
(PDF)

Figure S4 **Cells with low intracellular Mtb appear like uninfected cells.** BAL cells were isolated from WT mice 2 weeks p.i. and cytospin slides were prepared for (A) Ziehl-Neelsen or (B) DAPI plus carbolfuchsin staining. AFB were identified with light microscopy or fluorescence microscopy (magnification, 400×). Images of AFB^+^ cells with low intracellular Mtb appear similar in nuclear morphology with adjacent uninfected cells. Survey thousands of cells contain low number of bacilli identified none with the morphological features of necrosis that was typical of heavily infected cells.
(PDF)

Figure S5 **Chromatin extrusion from DAPI stained AFB^+^ cells.** BAL cells from mice with aerogenic TB infected were harvested 3 weeks, p.i. Samples were prepared on cytospin slides and stained with DAPI. The image shows nuclear condensation and chromatin extruding through a damaged nuclear membrane into the cytoplasm (*white arrow*; magnification, ×400).
(PDF)

Figure S6 **Morphology of neutrophil cell death in pulmonary TB.** (A) Ziehl-Neelsen staining (*left panel*) identified amorphous extracellular material with associated AFB (magnification, ×400). Scanning EM (*middle panel*) demonstrated the presence of cell-associated extracellular fibers consistent with NETs (magnification, ×5,000). High resolution of SEM image (*right panel*) revealed globular domains decorating the extracellular fibrous structures (magnification, ×25,000). (B) NETs were identified by immunostaining using DAPI to stain DNA (*blue*) and antibodies against histone H2B (*green*) and MPO (*red*) or neutrophil elastase (*green*) and MPO (red). Stained cells were analyzed using confocal scanning laser microscopy (magnification, 63× objective). (C) BAL cells from ApoE null mice 4 weeks p.i. were stained for DNA (*blue*), histone H2B (*green*) and MPO (*red*) and visualized with confocal scanning laser microscopy.
(PDF)

Figure S7 **Higher burst size parameter values result in higher total bacterial counts in computational simulation of Mtb replication in the lung.** A multiscale computational model described in *Materials and Methods* was used to generate values for total bacterial counts over time in a 2 mm×2 mm virtual section of lung starting with a single macrophage infected with a single bacillus at time zero. The different curves correspond to different burst size parameter values for the number of Mtb bacilli within a macrophage that induce cytolysis. All the other parameters in the computational model capturing immune mechanisms are identical for each curve and are calibrated to reproduce a typical chronic Mtb infection in a mouse. The x-axis shows days after time zero, while the y-axis shows mean total Mtb counts ± SD for 20 individual program runs at each burst size value. For ease of illustration, significant differences (p<0.05) are not shown on the graph. Overall, *t*-test results show that higher burst size values favor higher total lung bacterial load. Scaling to the whole lung can be done by multiplying the prediction by a factor of ∼10^4^, assuming a mouse lung volume of ∼1 cm^3^. The scaling returns CFU in the whole lung in the range of 1–10×10^6^.
(PDF)

Table S1 **Percentage and total cell count of different cells from lung leukocytes.**
(PDF)

Table S2 **Distribution of AFB^+^ cells for each cell type found in lung leukocytes.**
(PDF)

Table S3 **Percentage and total cell count of different GFP^+^ cells from lung leukocytes.**
(PDF)
