# Supplementary figures and images for: Intracellular Bacillary Burden Reflects a Burst Size for Mycobacterium tuberculosis In Vivo
Source: PLoS Pathog. 2013 Feb 21;9(2):e1003190. doi: 10.1371/journal.ppat.1003190 (PMC3578792; doi:10.1371/journal.ppat.1003190)

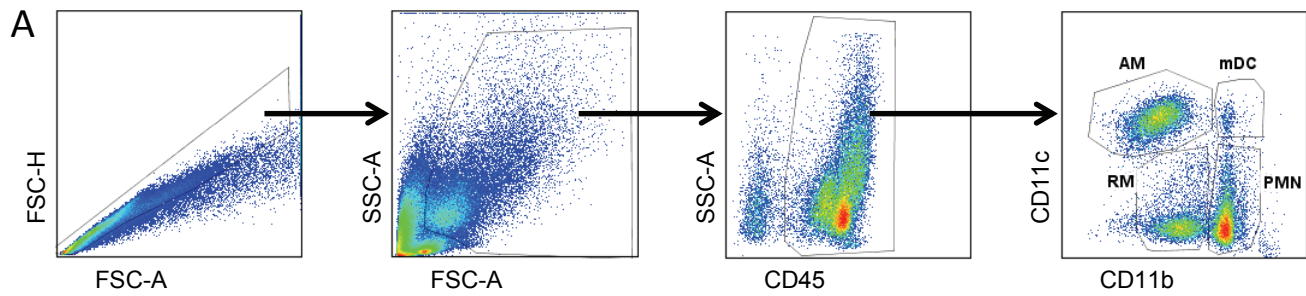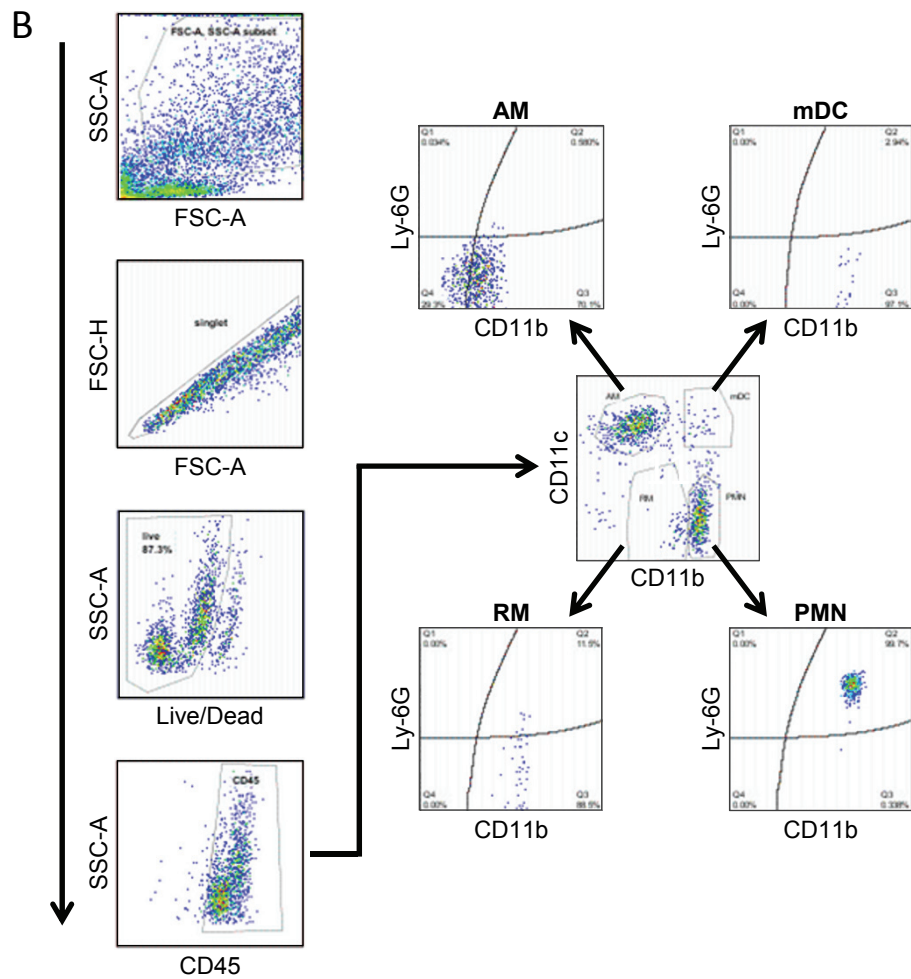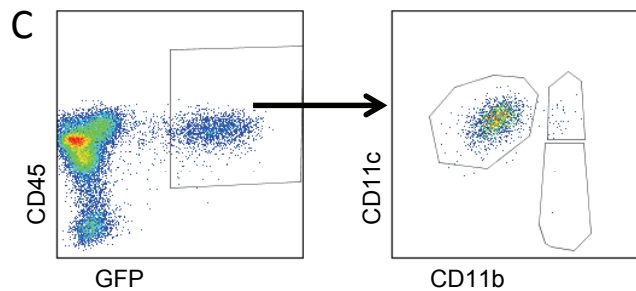

Supplement: Figure S1 — Gating scheme for immunophenotyping lung leukocyte populations. (A) Gating steps to analyze lung cell populations include gating on singlets as having a similar height and area (FSC-A/FSC-H), on live and myeloid cells (FSC-A/SSC-A), and on CD45+ hematopoietic cells (SSC-A/CD45). Each cell population is defined based on CD11b/CD11c staining profile: alveolar macrophages (AM, CD11b− CD11c+/hi), myeloid DC (mDC, CD11b+/hi CD11c+/hi), neutrophils (PMN, CD11b+/hi CD11c−), and recruited monocyte-macrophages (RM, CD11b+/lo CD11clo/−). (B) Schematic illustration of the gating strategy of lung leukocytes. A stepwise gating strategy was applied to lung cell populations gating on myeloid phagocytes, singlets, live cells, and CD45+ cells. CD45+ cells are divided into four cell types based on CD11b/CD11c expression profiles. Each cell type is further analyzed by CD11b/Ly-6G staining profiles. Curved quadrants were set according to fluorescence-minus-one. This figure indicates that the cell population defined by CD11b/CD11c profile is identical to those by CD11b/Ly-6G. (C) Gating on GFP expressing lentivirus-transduced leukocytes. Cells were transduced in vivo by tracheal instillation of CMV-GFP-W lentivirus as described in Materials and Methods. This approach exclusively labels cells within the airspace of the lung. After an 8 week rest period to allow the GFP+ cell population to stabilize, BAL cells were recovered for CD11b/CD11c immunostaining and flow cytometry. Based on a CD45/GFP scatter graph, GFP is expressed exclusively by CD45+ cells. Most of GFP+ cells comprise AM (CD11b− CD11c+/hi) consistent with the fact that the vast majority of leukocytes in the alveolar space under basal conditions are resident AM. A very small proportion of GFP+ cells fall in the mDC gate. (PDF) [file ppat.1003190.s001.pdf]

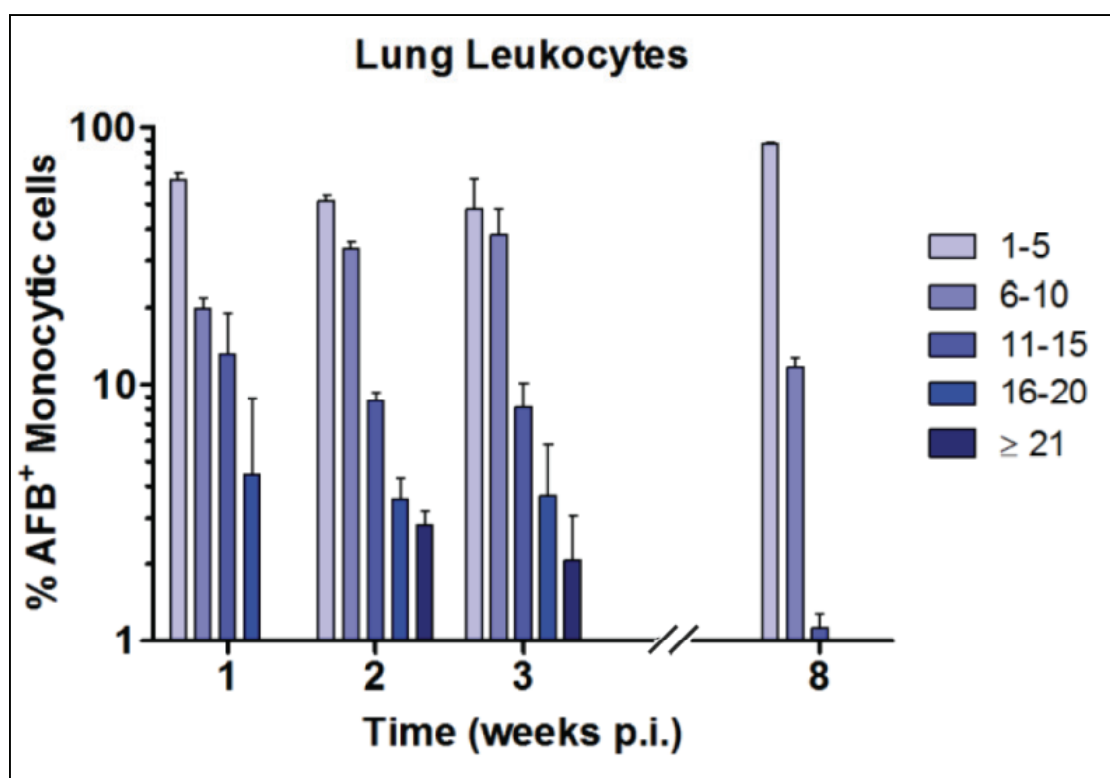

Supplement: Figure S2 — Change in the proportion of intracellular bacillary load in monocytic cells. AFB per cell was counted in cytospin samples of whole lung leukocytes harvested 1, 2, 3 and 8 weeks after aerosol challenge with Mtb Erdman. Mtb burden per monocytic cell (comprising AM, RM, mDC) was counted and stratified into the indicated bins of 1–5, 6–10, 11–15, 16–20, or ≥21. Results are expressed as mean % AFB+ monocytic cells within each bin ± SD at the indicated time points. Statistical analysis described in Materials and Methods confirmed a significantly different distribution of AFB load in high bins at 8 weeks p.i. as compared to earlier time points. (PDF) [file ppat.1003190.s002.pdf]

A

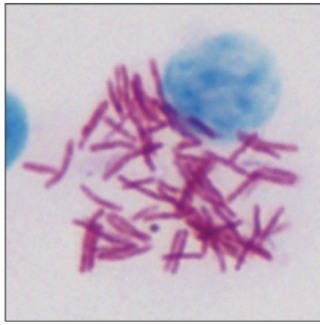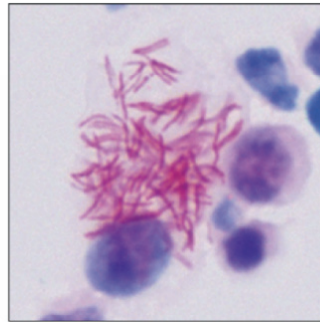

B

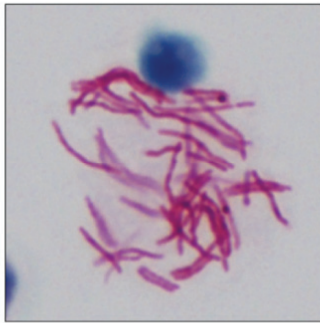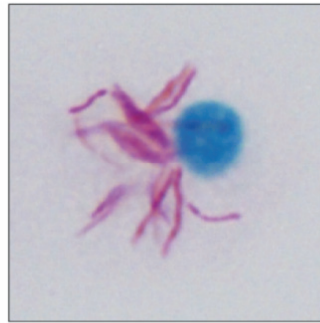

C

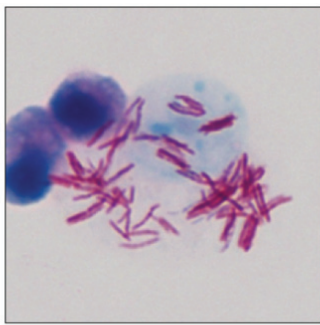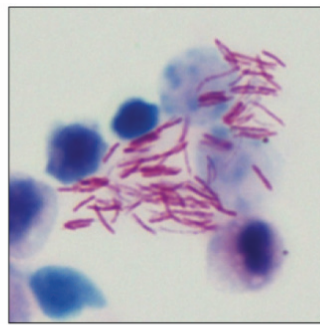

Supplement: Figure S3 — Cells heavily burdened with Mtb appear nonviable. (A) Lung leukocytes were isolated from WT mice 2 weeks after aerosol challenge with Mtb Erdman. Cytospin preparations were made and Ziehl-Neelsen stain was used to visualize and count intracellular AFB by light microscopy at 400× magnification. Photomicrographs show examples of heavily infected cells with ∼50 intracellular AFB. (B) Whole lung leukocytes harvested 4 weeks after aerosol Mtb challenge were prepared for cell sorting. Cytospin preparations were made from the sorted population of “dead” cells defined by lower forward-scatter and higher side-scatter characteristics. AFB where visualized with Ziehl-Neelsen staining (magnification, ×400). (C) Lung leukocytes from WT mice with 3 weeks of TB disease were processed by cytocentrifugation and Ziehl-Neelsen staining. The image shows clumps of AFB associated with dead cell remnants barely capable of retaining dye (magnification, ×400). (PDF) [file ppat.1003190.s003.pdf]

A

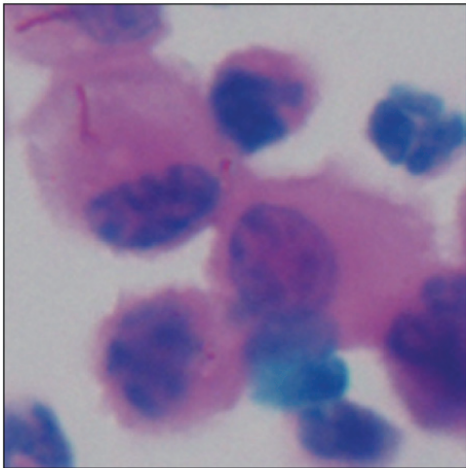

B

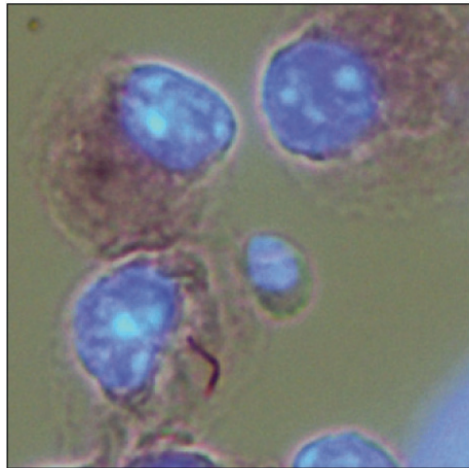

Supplement: Figure S4 — Cells with low intracellular Mtb appear like uninfected cells. BAL cells were isolated from WT mice 2 weeks p.i. and cytospin slides were prepared for (A) Ziehl-Neelsen or (B) DAPI plus carbolfuchsin staining. AFB were identified with light microscopy or fluorescence microscopy (magnification, 400×). Images of AFB+ cells with low intracellular Mtb appear similar in nuclear morphology with adjacent uninfected cells. Survey thousands of cells contain low number of bacilli identified none with the morphological features of necrosis that was typical of heavily infected cells. (PDF) [file ppat.1003190.s004.pdf]

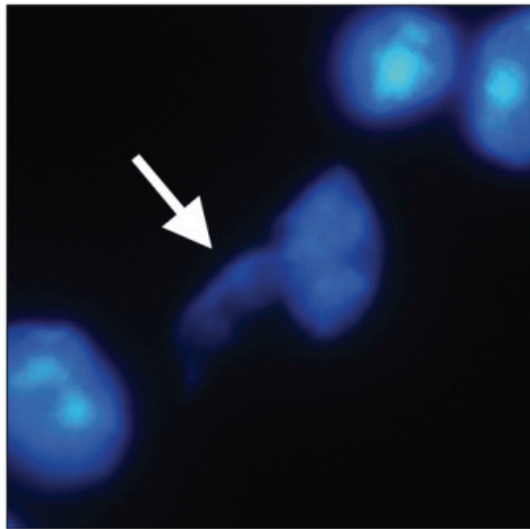

Supplement: Figure S5 — Chromatin extrusion from DAPI stained AFB+ cells. BAL cells from mice with aerogenic TB infected were harvested 3 weeks, p.i. Samples were prepared on cytospin slides and stained with DAPI. The image shows nuclear condensation and chromatin extruding through a damaged nuclear membrane into the cytoplasm (white arrow; magnification, ×400). (PDF) [file ppat.1003190.s005.pdf]

A

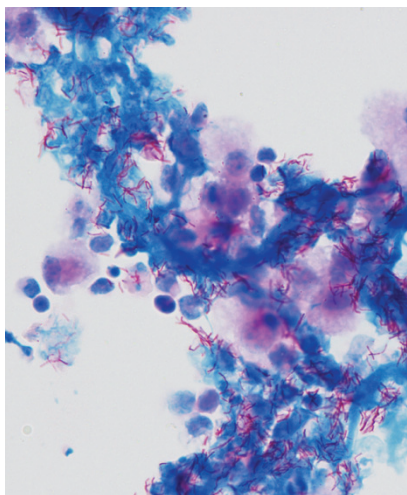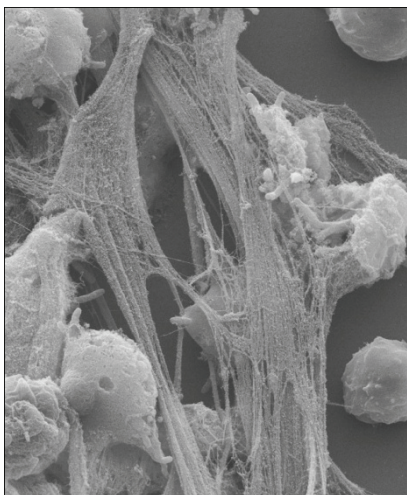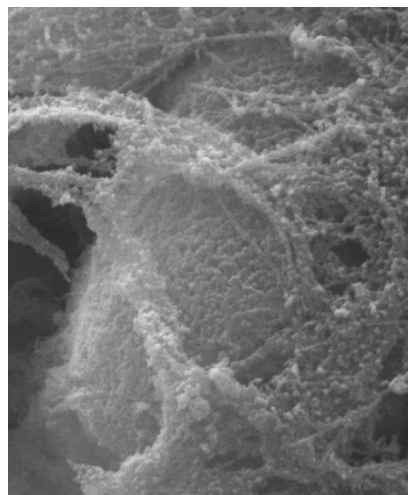

B

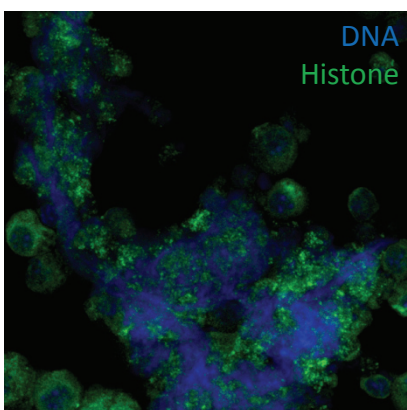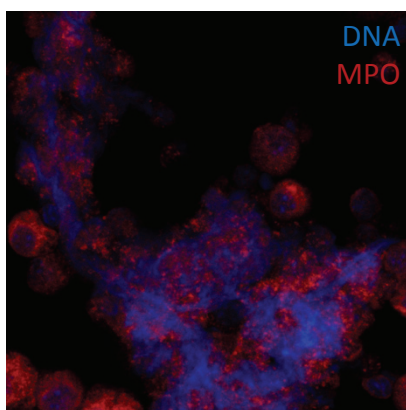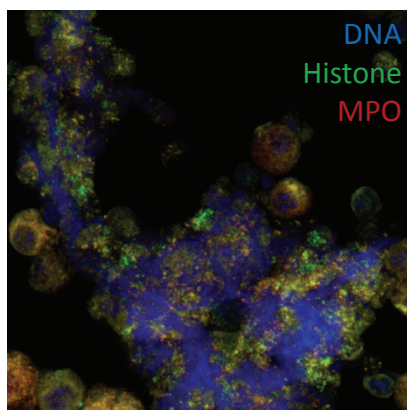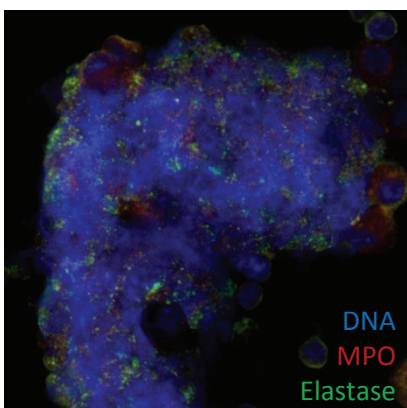

C

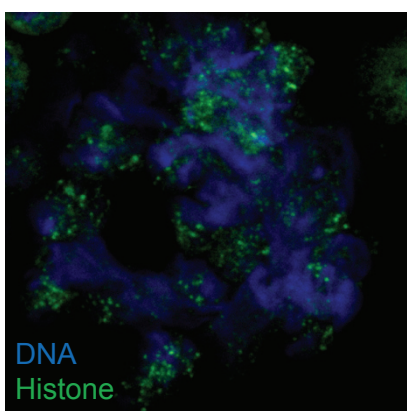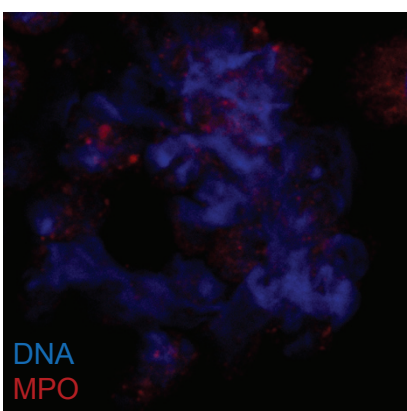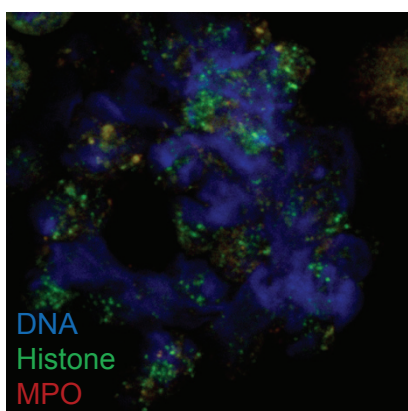

Supplement: Figure S6 — Morphology of neutrophil cell death in pulmonary TB. (A) Ziehl-Neelsen staining (left panel) identified amorphous extracellular material with associated AFB (magnification, ×400). Scanning EM (middle panel) demonstrated the presence of cell-associated extracellular fibers consistent with NETs (magnification, ×5,000). High resolution of SEM image (right panel) revealed globular domains decorating the extracellular fibrous structures (magnification, ×25,000). (B) NETs were identified by immunostaining using DAPI to stain DNA (blue) and antibodies against histone H2B (green) and MPO (red) or neutrophil elastase (green) and MPO (red). Stained cells were analyzed using confocal scanning laser microscopy (magnification, 63× objective). (C) BAL cells from ApoE null mice 4 weeks p.i. were stained for DNA (blue), histone H2B (green) and MPO (red) and visualized with confocal scanning laser microscopy. (PDF) [file ppat.1003190.s006.pdf]

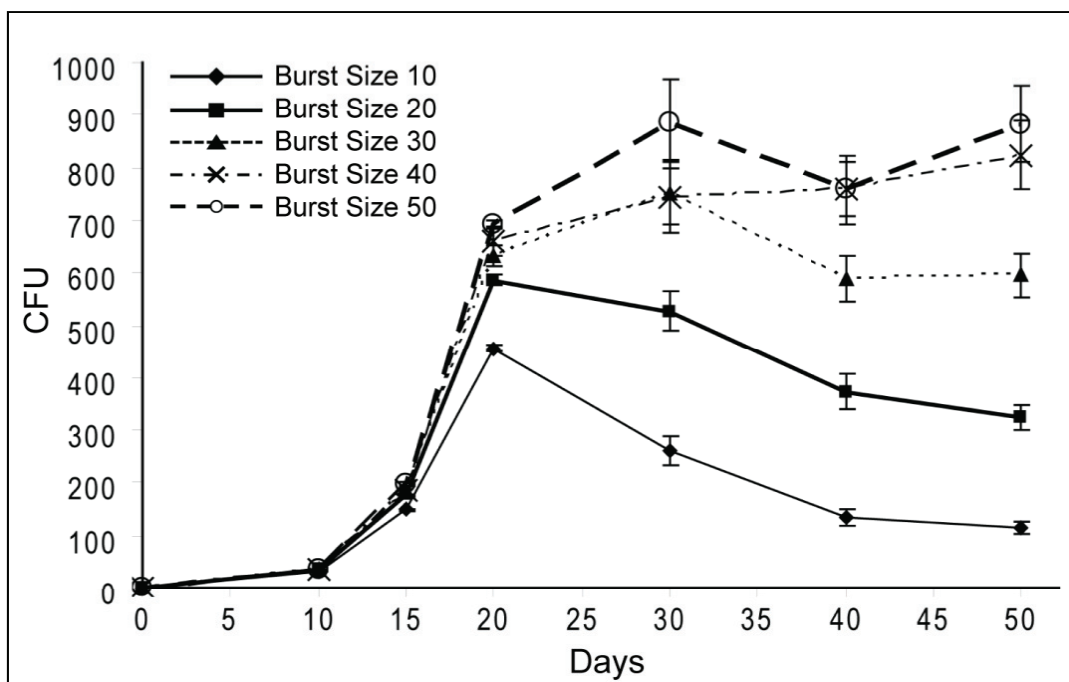

Supplement: Figure S7 — Higher burst size parameter values result in higher total bacterial counts in computational simulation of Mtb replication in the lung. A multiscale computational model described in Materials and Methods was used to generate values for total bacterial counts over time in a 2 mm×2 mm virtual section of lung starting with a single macrophage infected with a single bacillus at time zero. The different curves correspond to different burst size parameter values for the number of Mtb bacilli within a macrophage that induce cytolysis. All the other parameters in the computational model capturing immune mechanisms are identical for each curve and are calibrated to reproduce a typical chronic Mtb infection in a mouse. The x-axis shows days after time zero, while the y-axis shows mean total Mtb counts ± SD for 20 individual program runs at each burst size value. For ease of illustration, significant differences (p<0.05) are not shown on the graph. Overall, t-test results show that higher burst size values favor higher total lung bacterial load. Scaling to the whole lung can be done by multiplying the prediction by a factor of ∼104, assuming a mouse lung volume of ∼1 cm3. The scaling returns CFU in the whole lung in the range of 1–10×106. (PDF) [file ppat.1003190.s007.pdf]
