# Supplementary material for: Intracellular Bacillary Burden Reflects a Burst Size for Mycobacterium tuberculosis In Vivo
Source: PLoS Pathog. 2013 Feb 21;9(2):e1003190. doi: 10.1371/journal.ppat.1003190 (PMC3578792; doi:10.1371/journal.ppat.1003190)
Supplement: Table S1 — Percentage and total cell count of different cells from lung leukocytes. (PDF) [file ppat.1003190.s008.pdf]

Table S1. Percentage and total cell count of different cells from lung leukocytes.†

	Uninfected		4 weeks p.i.		Uninfected		10 weeks p.i.	
	Cells x 10^4	%	Cells x 10^4	%	Cells x 10^4	%	Cells x 10^4	%
AM	14.1 ± 3.4	41.9	47.8 ± 15.9*	34.9	16.6 ± 2.6	22.8	45.6 ± 9.4*	27.8
mDC	0.8 ± 0.4	1.6	25.3 ± 7.7*	18.5	0.4 ± 0.1	0.6	20.8 ± 6.8*	12.7
RM	15 ± 3.7	56.4	63.7 ± 5.8*	46.6	26.3 ± 6.5	36.2	97.5 ± 13.6*	59.5
Total	29.9		136.8		43.3		163.9	

†Results are presented as mean ± SD. *Indicates statistical significance.
