# Supplementary material for: Intracellular Bacillary Burden Reflects a Burst Size for Mycobacterium tuberculosis In Vivo
Source: PLoS Pathog. 2013 Feb 21;9(2):e1003190. doi: 10.1371/journal.ppat.1003190 (PMC3578792; doi:10.1371/journal.ppat.1003190)
Supplement: Table S2 — Distribution of AFB+ cells for each cell type found in lung leukocytes. (PDF) [file ppat.1003190.s009.pdf]

Table S2. Distribution of AFB$^+$ cells for each cell type found in lung leukocytes.

	4 weeks p.i.				10 weeks p.i.			
	Cells x 10^4	%	AFB$^+$ cells	%	Cells x 10^4	%	AFB$^+$ cells	%
AM	47	34.8	0.02	10.9	45	27.8	0.1	8.4
mDC	25	18.5	0.14	78.2	20	12.3	0.37	31.2
RM	63	46.7	0.02	10.9	97	59.9	0.71	60.4
Total	135		0.17		162		1.18	
