# Supplementary material for: Intracellular Bacillary Burden Reflects a Burst Size for Mycobacterium tuberculosis In Vivo
Source: PLoS Pathog. 2013 Feb 21;9(2):e1003190. doi: 10.1371/journal.ppat.1003190 (PMC3578792; doi:10.1371/journal.ppat.1003190)
Supplement: Table S3 — Percentage and total cell count of different GFP+ cells from lung leukocytes. (PDF) [file ppat.1003190.s010.pdf]

Table S3. Percentage and total cell count of different GFP⁺ cells from lung leukocytes.†

	Uninfected		4 weeks p.i.		Uninfected		10 weeks p.i.	
	Cells x 10^4	%	Cells x 10^4	%	Cells x 10^4	%	Cells x 10^4	%
AM	3.5 ± 0.9	97	2.28 ± 1.4	68.1	4.1 ± 0.8	97.9	2 ± 0.6*	75.2
mDC	0.1 ± 0.1	2.8	0.9 ± 0.6*	26.9	0.08 ± 0.04	1.9	0.6 ± 0.08*	22.6
RM	0.003 ± 0.0	0.1	0.047 ± 0.0*	1.4	0.002 ± 0.0	0.05	0.02 ± 0.01*	0.8
Other	0.006 ± 0.0	0.2	0.12 ± 0.02*	3.6	0.005 ± 0.0	0.1	0.04 ± 0.02*	1.5
Total	3.7		3.3		4.2		2.6	

†Results are presented as mean ± SD. *Indicates statistical significance.
